# The potential of a multimodal digital care program in addressing healthcare inequities in musculoskeletal pain management

**DOI:** 10.1038/s41746-023-00936-2

**Published:** 2023-10-10

**Authors:** Anabela C. Areias, Maria Molinos, Robert G. Moulder, Dora Janela, Justin K. Scheer, Virgílio Bento, Vijay Yanamadala, Steven P. Cohen, Fernando Dias Correia, Fabíola Costa

**Affiliations:** 1 Sword Health, Inc, Draper, UT USA; 2 Institute for Cognitive Science, University of Colorado Boulder, Boulder, CO USA; 3 Department of Neurological Surgery, University of California, San Francisco, CA USA; 4 Department of Surgery, Quinnipiac University Frank H. Netter School of Medicine, Hamden, CT USA; 5 Department of Neurosurgery, Hartford Healthcare Medical Group, Westport, CT USA; 6 Department of Anesthesiology & Critical Care Medicine, Physical Medicine and Rehabilitation, Neurology, and Psychiatry and Behavioral Sciences, Johns Hopkins School of Medicine, Baltimore, MD USA; 7 Department of Anesthesiology and Physical Medicine and Rehabilitation, Uniformed Services University of the Health Sciences, Bethesda, MD USA; 8 Neurology Department, Centro Hospitalar e Universitário do Porto, Porto, Portugal

**Keywords:** Rehabilitation, Pain management

## Abstract

Digital interventions have emerged as a solution for time and geographical barriers, however, their potential to target other social determinants of health is largely unexplored. In this post-hoc analysis, we report the impact of social deprivation on engagement and clinical outcomes after a completely remote multimodal musculoskeletal (MSK) digital care program managed by a culturally-sensitive clinical team. Patients were stratified in five categories according to their social deprivation index, and cross-referenced with their race/ethnicity, rurality and distance to healthcare facilities. From a total of 12,062 patients from all U.S. states, 8569 completed the program. Higher social deprivation was associated with greater baseline disease burden. We observed that all categories reported pain improvements (ranging from −2.0 95%CI −2.1, −1.9 to −2.1 95%CI −2.3, −1.9, *p* < 0.001) without intergroup differences in mean changes or responder rates (from 59.9% (420/701) to 66.6% (780/1172), *p* = 0.067), alongside reduction in analgesic consumption. We observed significant improvements in mental health and productivity across all categories, with productivity and non-work-related functional recovery being greater within the most deprived group. Engagement was high but varied slightly across categories. Together these findings highlight the importance of a patient-centered digital care program as a tool to address health inequities in musculoskeletal pain management. The idea of investigating social deprivation within a digital program provides a foundation for future work in this field to identify areas of improvement.

## Introduction

Healthy People 2030 initiative states that *health equity* “requires valuing everyone equally (…) to address avoidable inequalities, historical and contemporary injustices, and social determinants of health (SDH)—and to eliminate disparities in health and health care.”^1^ Whereas some attribute access to healthcare as the main barrier, social determinants of health research has identified other non-medical factors as important health drivers^2^. Living location, education, economic stability, and cultural and community context account for nearly 40% of a person’s health status^2^. These factors can impact on patients’ healthcare timely access, trust (and respective compliance) and affordability. In musculoskeletal (MSK) care, poverty, low education levels, and being people of color have been associated with a higher prevalence of MSK complaints, poorer function, and greater pain severity. Only some of which can be explained by access to healthcare^3–5^, highlighting the importance of a holistic patient-centered approach.

MSK pain management guidelines primarily focus on exercise-based interventions^6–9^. However, poor access to providers, schedule constraints, pressure to work when sick, travel and absenteeism costs and inadequate health literacy, in addition to other SDH, have hindered the access of millions to proper care^10^. Timely access to care may reduce the need for treatment escalation, minimizing the use of low value care (e.g., unnecessary injections, surgeries), and preventing opioid use^10,11^.

To address geographical and financial barriers, stakeholders must recognize the unique needs of historically marginalized groups. Clinical teams should be culturally diverse, proficient in different languages and have specific training to minimize the potential for discrimination and implicit bias^12–14^. Telerehabilitation has emerged as an effective solution for MSK pain management^15^, as it increases access and convenience, fills gaps in provider coverage (particularly in underserved areas), and improves care continuity. However, the impact of telerehabilitation in addressing SDH to promote health equity is an unexplored area.

The present study reports upon a patient-centered digital care program (DCP) combining exercise with education and cognitive behavioral therapy (CBT), which was designed to promote health equity by eliminating geographic and time barriers, while providing a diverse pool of physical therapists in terms of gender, race/ethnicity, cultural background and spoken languages.

In the past we have validated this DCP for several acute and chronic MSK conditions^16–19^, and reported on its effectiveness regardless of race/ethnicity^20^ and geographical location (rural vs urban)^21^, suggesting its potential in mitigating some health inequities. The present study applies a social deprivation index (SDI) to a broad cohort of patients with chronic MSK pain who underwent this DCP, aiming to assess the impact of socioeconomic context on clinical outcomes and engagement. This post hoc analysis hypothesizes that categories from different socioeconomic contexts would experience similar significant improvements in outcomes.

## Results

**Fig. 1:**
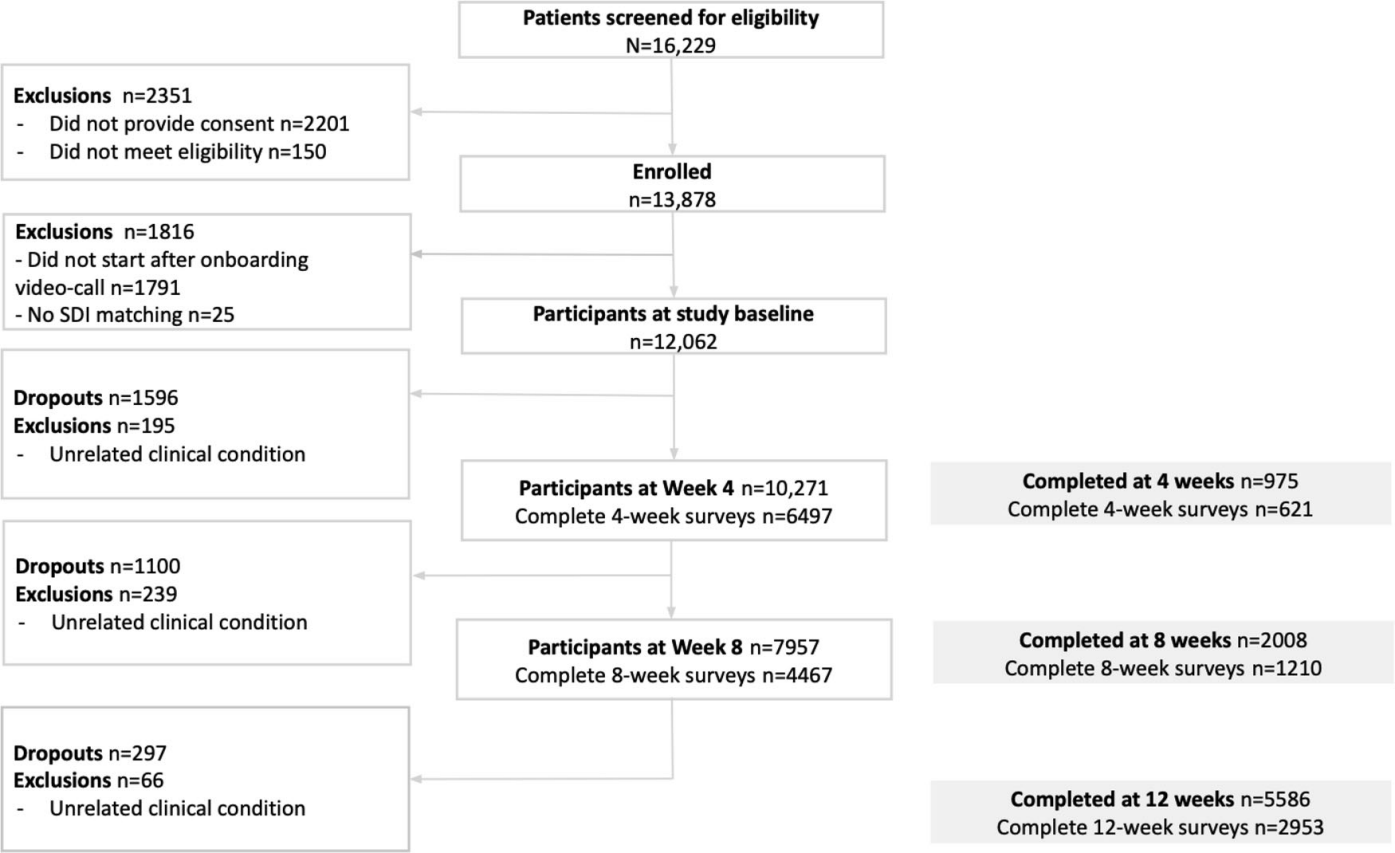
Flowchart of the study following the CONSORT (Consolidated Standards of Reporting Trials) guidelines. Abbreviations: SDI Social Deprivation Index.

From a total of 16,229 participants screened for eligibility, 12,062 patients (74.3%) started the study as depicted in Fig. 1. Among these, a total of 8569 (71.0%) patients completed the program. Patients were assigned to a specific SDI based on their ZIP codes and then categorized into fifths using SDI as indexing variable. Completion rates were high across SDI categories, with dropout rates varying linearly with increased category (assessed through a binary logistic regression): C1 22.1% (810/3666); C2 23.1% (669/2903); C3 26.7% (639/2398); C4 27.9% (522/1874); C5 28.9% (353/1221), (OR *p* values: 1.23, <0.001 (1st degree); 0.95, 0.298 (2nd degree); 0.95, 0.270 (3rd degree); 1.05, 0.354 (4th degree)).

### Baseline demographic characteristics

**Fig. 2:**
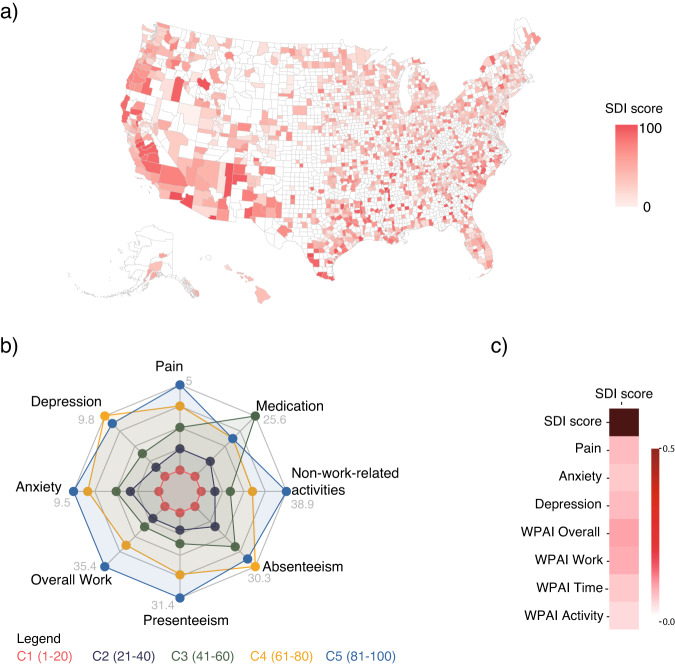
Baseline characteristics. **a** Heatmap of patient’s location per SDI score. **b** Baseline clinical scores across the SDI categories: radar chart (higher distance from the center represents a higher mean or proportion (worse score)). **c** Spearman correlation between clinical scores and SDI scores (as continuous variable). For all correlations, *p* values were below the 0.01 significance level; darker red color denotes higher correlation, with correlation between both SDI scores being equal to 1.

The cohort distribution across the U.S. (Fig. 2a) included 3666 patients in category 1 (C1), 2903 in C2, 2372 in C3, 1874 in C4 and 1221 in C5 (C5 corresponding to areas with higher social deprivation). Demographic characteristics varied across SDI categories (Table 1). Those within C5 were younger (mean 45.7 years, SD 11.5, *p* < 0.001), and had a higher body mass index (BMI, mean 30.1, SD 7.0, *p* < 0.001). There were higher proportions of patients of Black (20.3%, *p* < 0.001) and Hispanic (15.4%, *p* < 0.001) background, as well as fewer people with higher education (16.5%, *p* < 0.001) in C5. No changes were observed in employment rates between groups (*P* = 0.357). Affected anatomical areas were similarly distributed across categories, except for the knee (C1 and C2 vs C3, C4 and C5, *p* = 0.014). Back pain was the most prevalent condition in all categories. All categories had patients primarily located in urban areas, with C1 and C5 having the lowest proportion residing in rural areas. Patients in C5 lived closer to healthcare facilities (median 1.7 miles IQR 2.6, *p* < 0.001) than those in other categories and had the highest number of available providers within 18 miles of their residence (median 37 IQR 85, *p* < 0.001).

**Table 1:** Baseline characteristics by Social Deprivation Index categories^a^.

Characteristic	Category 1 (lowest)	Category 2	Category 3	Category 4	Category 5 (highest)	*p* value
Number (#,%)	3666 (30.4)	2903 (24.1)	2398 (19.9)	1874 (15.5)	1221 (10.1)	<0.001
Age in years (mean, SD)	49.3 (11.0)	48.3 (11.5)	47.6 (11.8)	47.8 (11.7)	45.7 (11.5)	<0.001
Age Category (#,%)						
<25 years	43 (1.2)	30 (1.0)	35 (1.5)	24 (1.3)	19 (1.6)	<0.001
25–40	809 (22.1)	758 (26.1)	711 (29.6)	551 (29.4)	436 (35.7)	
40–60	2171 (59.2)	1606 (55.3)	1244 (51.9)	976 (52.1)	621 (50.9)	
>60	643 (17.5)	509 (17.5)	408 (17.0)	323 (17.2)	145 (11.9)	
Gender (#,%)						
Woman	1998 (54.5)	1738 (59.9)	1385 (57.8)	1073 (57.3)	741 (60.7)	0.006
Man	1654 (45.1)	1156 (39.8)	1006 (42)	791 (42.2)	473 (38.7)	
Non-binary	11 (0.3)	6 (0.2)	7 (0.3)	7 (0.4)	6 (0.5)	
Other	1 (0)	0 (0)	0 (0)	1 (0.1)	0 (0)	
Prefers not to answer	2 (0.1)	3 (0.1)	0 (0)	2 (0.1)	1 (0.1)	
BMI (mean, SD)^b^	28.5 (6.2)	28.9 (6.7)	29.4 (6.9)	29.8 (6.7)	30.1 (7.0)	<0.001
BMI Category (#,%)						
Underweight	37 (1)	20 (0.7)	22 (0.9)	11 (0.6)	8 (0.7)	<0.001
Normal	1131 (30.9)	886 (30.6)	643 (26.9)	450 (24.1)	290 (23.8)	
Overweight	1294 (35.4)	951 (32.8)	809 (33.8)	629 (33.6)	395 (32.4)	
Obese	1001 (27.4)	831 (28.7)	714 (29.8)	634 (33.9)	406 (33.3)	
Morbidly obese	195 (5.3)	208 (7.2)	206 (8.6)	146 (7.8)	122 (10)	
Race and Ethnicity (#,%)						
Asian	342 (9.3)	253 (8.7)	148 (6.2)	142 (7.6)	61 (5.0)	<0.001
Black	142 (3.9)	168 (5.8)	171 (7.1)	207 (1.1)	248 (20.3)	
Hispanic	171 (4.7)	176 (6.1)	207 (8.6)	191 (10.2)	188 (15.4)	
Non-Hispanic White	1909 (52.1)	1532 (52.8)	1188 (49.5)	818 (43.6)	404 (33.1)	
Other	53 (1.4)	58 (2)	55 (2.3)	46 (2.5)	36 (2.9)	
Not available/Prefers not specify	1049 (28.6)	716 (24.7)	629 (26.2)	470 (25.1)	284 (23.3)	
Employment status (#,%)						
Employed	3325 (90.7)	2656 (91.5)	2152 (89.7)	1721 (91.8)	1118 (91.6)	0.357
Not Employed	253 (6.9)	178 (6.1)	182 (7.6)	115 (6.1)	73 (6)	
Not available/Prefers not to answer	88 (2.4)	69 (2.4)	64 (2.7)	38 (2)	30 (2.5)	
Education level (#,%)						
Less than high school diploma	16 (0.4)	18 (0.6)	10 (0.4)	21 (1.1)	15 (1.2)	<0.001
High school diploma	175 (4.8)	180 (6.2)	195 (8.1)	158 (8.4)	127 (10.4)	
Some college	583 (15.9)	603 (20.8)	562 (23.4)	455 (24.3)	303 (24.8)	
Bachelor’s degree	1389 (37.9)	1010 (34.8)	750 (31.3)	550 (29.3)	380 (31.1)	
Graduate degree	869 (23.7)	631 (21.7)	488 (20.4)	380 (20.3)	202 (16.5)	
Prefers not to answer/Not available	634 (17.3)	461 (15.9)	393 (16.4)	310 (16.5)	194 (15.9)	
Anatomical region affected (#,%)						
Ankle	148 (4)	118 (4.1)	115 (4.8)	82 (4.4)	48 (3.9)	0.014
Elbow	104 (2.8)	71 (2.4)	48 (2)	31 (1.7)	20 (1.6)	
Hip	366 (10)	292 (10.1)	245 (10.2)	182 (9.7)	92 (7.5)	
Knee^c^	527 (14.4)	421 (14.5)	369 (15.4)	301 (16.1)	231 (18.9)	
Low back	1387 (37.8)	1087 (37.4)	898 (37.4)	714 (38.1)	467 (38.2)	
Neck	387 (10.6)	296 (10.2)	265 (11.1)	179 (9.6)	126 (10.3)	
Shoulder	627 (17.1)	499 (17.2)	378 (15.8)	310 (16.5)	187 (15.3)	
Wrist/hand	120 (3.3)	119 (4.1)	80 (3.3)	75 (4)	50 (4.1)	
Geographic location (#,%)						
Urban	3494 (95.3)	2564 (88.3)	1993 (83.1)	1551 (82.8)	1114 (91.2)	<0.001
Rural	172 (4.7)	339 (11.7)	405 (16.9)	323 (17.2)	107 (8.8)	
Minimum distance to nearest healthcare facilities in miles^d^						
Median (IQR)	2.7 (3.7)	2.4 (4.1)	2.4 (4.4)	2.2 (4.3)	1.7 (2.6)	<0.001
Number of healthcare facilities within, median (IQR):^d^						
6 miles	3.0 (7)	4.0 (10)	4.0 (11)	4.0 (12)	7.0 (18)	<0.001
18 miles	33.0 (44)	27.0 (59)	21.0 (58)	22.0 (68)	37.0 (85)	<0.001

*BMI* body mass index, *GAD-7* Generalized Anxiety Disorder 7-item scale, *PHQ-9* Patient Health 9-item questionnaire, *WPAI* Work Productivity and Activity Impairment questionnaire.

^a^Data represents mean ± standard deviation or the number of patients and % of total where listed. *p-*values represent comparison between SDI categories through 1-way ANOVA or Pearson Chi-square test, except those indicated otherwise.

^b^23 missing values.

^c^C1 versus C4 was the only statistically significant identified through Bonferroni post hoc analysis, *p* = 0.014.

^d^Comparisons were performed through Kruskal-Wallis test.

### Baseline clinical characteristics

Baseline clinical outcomes across SDI categories are presented in Fig. 2b and in Supplementary Table 1. Overall, patients in C5 reported a higher disease burden, with higher levels of pain (5.0, SD 2.0, *p* < 0.001), anxiety (9.5, SD 4.5, *p* < 0.001), and productivity impairment (WPAI overall: 35.4, SD 23.8, *p* < 0.001; WPAI work: 31.4, SD 20.1, *p* < 0.001; and WPAI activity: 38.9, SD 23.4, *p* = 0.005). Higher social deprivation was associated with worse baseline clinical scores (*p* < 0.001 all, Fig. 2c).

### Clinical outcomes

Clinical outcomes are presented in Table 2 (entire cohort analysis available in Supplementary Table 2). LGCA models yielded good fit (model estimates and fitness are presented in Supplementary Tables 3 and 4, respectively).

**Table 2:** Program-end and estimated outcome mean change for each Social Deprivation Index category^a^.

Outcome	Time	Category 1 (lowest)	Category 2	Category 3	Category 4	Category 5 (highest)
Pain Level	Program end	2.6 (2.5, 2.8)	2.6 (2.5, 2.8)	2.7 (2.6, 2.9)	2.8 (2.6, 2.9)	2.9 (2.7, 3.1)
	Mean Change	−2.0 (−2.1, −1.9)	−2.1 (−2.2, −1.9)	−2.1 (−2.2, −1.9)	−2.1 (−2.2, −1.9)	−2.1 (−2.3, −1.9)
	Response rate	64.8%	66.6%	63.8%	59.9%	63.4%
GAD-7 ≥ 5	Program end	4.0 (3.6, 4.4)	4.3 (3.8, 4.8)	4.9 (4.3, 5.5)	4.9 (4.3, 5.5)	5.0 (4.2, 5.7)
	Mean Change	−4.3 (−4.7, −3.9)	−4.4 (−4.8, −3.9)	−3.9 (−4.5, −3.4)	−4.4 (−4.9, −3.8)	−4.5 (−5.2, −3.8)
PHQ-9 ≥ 5	Program end	4.1 (3.6, 4.8)	4.4 (3.8, 5.0)	4.7 (4.0, 5.3)	5.1 (4.3, 5.8)	5.1 (4.2, 6.0)
	Mean Change	−4.8 (−5.3, −4.2)	−4.7 (−5.3, −4.1)	−4.6 (−5.2, −3.9)	−4.8 (−5.4, −3.9)	−4.6 (−5.5, −3.7)
WPAI - Overall > 0	Program end	14.7 (13.0, 16.4)	16.9 (14.9, 18.9)	16.3 (14.0, 18.6)	18.4 (15.9, 20.9)	16.2 (12.9, 19.5)
	Mean Change	−14.1 (−15.8, −12.3)	−13.8 (−15.2, −11.1)	−14.7 (−17.0, −12.4)	−14.5 (−17.2, −11.9)	−19.2 (−22.6, −15.9)
WPAI - Work > 0	Program end	12.9 (11.4, 14.4)	14.7 (12.9, 16.5)	13.2 (−11.3, −15.1)	16.0 (−13.7, −18.2)	14.8 (−12.0, −17.7)
	Mean Change	−13.9 (−15.5, −12.4)	−13.0 (−14.8, −11.2)	−15.3 (−17.2, −13.4)	−14.1 (−16.4, −11.7)	−16.5 (−19.4, −13.5)
WPAI - Time > 0	Program end	5.9 (3.0, 8.9)	8.3 (4.8, 11.8)	11.8 (5.9, 17.9)	7.3 −3.6, 11.0)	9.8 (−3.5, −16.1)
	Mean Change	−14.9 (−18.8, −11.0)	−15.5 (−19.7, −11.3)	−15.1 (−21.1, −9.0)	−23.0 (−28.9, −17.0)	−19.6 (−26.5, −12.6)
WPAI - Activity > 0	Program end	17.0 (15.9, 18.4)	18.1 (16.6, 19.5)	17.1 (15.5, 18.8)	17.8 (16.0, 19.7)	17.3 (15.0, 19.7)
	Mean Changes	−16.6 (−17.9, −15.3)	−16.5 (−18.0, −15.0)	−18.3 (−19.9, −16.6)	−18.9 (−20.8, −17.0)	−21.4 (−23.7, −19.0)

*GAD-7* Generalized Anxiety Disorder 7-item scale, *PHQ-9* Patient Health 9-item questionnaire, *WPAI* Work Productivity and Activity Impairment questionnaire.

^a^Intention-to-treat analysis. All within-group mean changes were significant at a level of *p* < 0.001 assessed by a multiple-group latent growth curve analysis. Response rate for pain was not significantly different between categories (*p* = 0.067) assessed using a binary logistic regression. Data represents mean (95% Confidence Intervals).

**Fig. 3:**
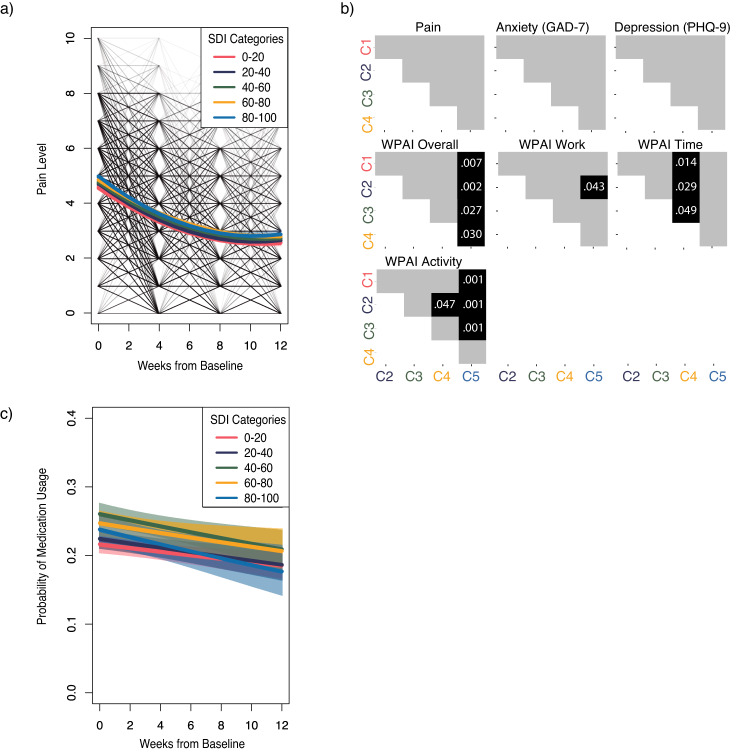
Clinical outcomes. **a** Pain trajectories per category. Shadowing indicates each trajectory confidence interval, while individual trajectories are depicted with lighter gray lines. **b** Heatmap depicting statistical significance for all outcomes across categories assessed by a multiple-group latent growth curve analysis (gray denotes *p* values > 0.05). **c** Medication reduction trajectories per category. Shadowing indicates each trajectory confidence interval.

Significant improvements were observed in all clinical outcomes across SDI categories (Table 2). Significant pain improvement was observed across categories, without intergroup statistical differences (Fig. 3a, b). A similar response rate for pain was observed in all categories (59.9% (420/701) in C4 to 66.6% (780/1172) in C1, *p* = 0.067) translating into nonlinear odds ratios between groups (*p* values: 0.060 (1st degree), 0.915 (2nd degree), 0.022 (3rd degree), 0.760 (4th degree)).

The proportion of patients taking pain medication was reduced by program-end in all SDI categories (Fig. 3c, Supplementary Table 5).

Significant and similar improvements in anxiety and depression (Table 2, Fig. 3b) were observed across categories for those with at least mild symptomatology at baseline.

Productivity impairment improved significantly in all WPAI domains across SDI categories (Table 2). Improvements in absenteeism were especially high, with reductions ranging from −14.9 (95%CI: −18.8, −11.0) to −23.0 (95%CI: −28.9, −17.0) (corresponding to a 55.9–75.8% change). Patients from C5 reported significantly greater changes in both impairment of overall productivity and non-work-related activities compared to other categories (Fig. 3b).

### Impact of covariates

Patients with higher BMI presented higher baseline pain, depression, overall productivity, and activity impairment regardless of the category (*p* values < 0.05, Supplementary Table 6). Women also had worse baseline pain, anxiety, depression and activity impairment across all categories, with overall productivity being affected in C1–C3 (Supplementary Table 6). Patients from Black and Hispanic backgrounds reported worse baseline pain in all categories, and productivity and activities impairment in some categories (Supplementary Table 6). The impact of covariates on recovery pace was not so evident, with race/ethnicity influencing some but not all categories in: pain (C5: faster recovery in Black and Hispanic patients), and non-work-related activities (faster recovery of Asians in C3 and of Black patients in C4), (reference category: non-Hispanic Whites; Supplementary Table 6). Those with higher BMI improved on depression at a faster pace compared to those with lower BMI in C1, C2, and C5.

### Engagement and satisfaction

Patient engagement was high across SDI categories (Table 3). The median total number of sessions performed in categories 1–5 were respectively 23.0 (IQR 35.0), 22.0 (IQR 35.0), 20.0 (IQR 35.0), 19.0 (IQR 36.0), and 17.0 (IQR 30.0), respectively (*p* < 0.001). Engagement with exercise sessions was impacted by gender, BMI, race/ethnicity and rurality. Patients with high BMI (all categories), women (C1 and C2) and patients from Asian (C3), Hispanic (C1 and C3) and Black (C1) backgrounds dedicated less time to exercise, whereas those in rural areas (C2 and C3) dedicated more time (for *p* values consult Supplementary Table 7).

**Table 3:** Patient Engagement stratified by Social Deprivation Index category.

Engagement outcomes	Category 1	Category 2	Category 3	Category 4	Category 5	*p* value^a^	Entire cohort
Total number of sessions, median (IQR), [min;max]	23.0 (35.0) [3;550]	22.0 (35.0) [3;434]	20.0 (35.0) [3;350]	19.0 (36.0) [3;280]	17.0 (30.0) [3;389]	<0.001	21.0 (34.0) [3;550]
Total training time, median (IQR), [min;max]	297.0 (494.8) [12.0;5715.9]	289.0 (490.0) [12.6;5728.3]	257.0 (477.8) [11.0;4842.2]	239.6 (472.4) [11.4;4397.4]	216.2 (398.1) [17.6;5421.6]	<0.001	268.1 (477.7) [11.0;5741.0]
Number of sessions per week, median (IQR), [min;max]	2.5 (1.5) [1.0;7.09]	2.5 (1.5) [1.0;9.3]	2.4 (1.5) [1.0;8.87]	2.4 (1.5) [1.0;8.9]	2.3 (1.3) [1.0;7.2]	<0.001	2.4 (1.5) [1.0;9.3]
Total articles read, median (IQR), [min;max]	1.0 (3.0) [0;44]	1.0 (3.0) [0;41]	1.0 (3.0) [0;58]	1.0 (3.0) [0;40]	1.0 (3.0) [0;47]	0.400	1.0 (3.0) [0;58]
Total interactions with the PT, median (IQR), [min;max]	10.0 (19.0) [0;105]	10.0 (18.0) [0;116]	9.0 (17.0) [0;139]	9.0 (18.0) [0;127]	10.0 (16.0) [0;146]	0.086	10.0 (17.0) [0;146]

^a^Kruskal-Wallis test.

Patients had a similar number of interactions with the DPT and read similar amounts of educational pieces.

Similar satisfaction scores were observed independently of the category: C1 8.9, SD 1.4; C2 8.9, SD 1.5; C3 8.9, SD 1.5; C4 9.0, SD 1.5; C5 9.0, SD 1.4; *p* = 0.542 (entire cohort: 8.9, SD 1.4).

## Discussion

The DCP was able to reach a wide range of SDI scores. Most patients fell into categories of low social deprivation, however 10% had high SDI scores, in line with the U.S. Census Bureau report^22^. As previously reported, SDI categories were associated with a growing severity of baseline disease burden, highlighting the existence of particularly vulnerable subpopulations^5,23,24^. These initial scores were not explained by distance to healthcare facilities since patients within the highest deprived category were also the ones geographically closest to the facilities. Independently of SDI, specific demographics were associated with aggravated baseline scores namely women^25^, overweight people^26^ and those who identified as Black and Hispanic backgrounds^3^. Altogether, these factors have motivated new healthcare models to evolve towards a holistic patient-centered care, as the DCP described herein. Low socioeconomic status was reported to be associated with worse treatment outcomes^27^ in opposition to the results herein observed, which showed a similar recovery path in all clinical outcomes regardless of SDI scores and baseline severity. Particularly for pain, the high and similar improvements and response rates contrast with prior reports, where the most socially deprived individuals attained worse outcomes in pain compared to their counterparts^27–29^. The observed improvements were in line with those reported previously after in-person physical therapy for patients with chronic pain^30,31^. Pain improvement was accompanied by a reduction in analgesic consumption. Early access to physical therapy has been reported to prevent chronic use of opioids, a potential solution for what has been a national crisis^32^.

Mental health is one pivotal factor in MSK pain management considering the frequent comorbidity and feedback loop^33^ exacerbated by SDH^23,24^. Significant improvements were observed in mental health outcomes, similarly between SDI categories, further supporting multimodal biopsychosocial approaches^34^.

Literature evaluating the impact of SDH in the recovery of mental health in patients with MSK conditions is still lacking, with in-person or telerehabilitation interventions reporting highly heterogeneous results in this domain. Nevertheless, the significant changes observed in both mental health outcomes were within the range of the best improvements previously reported in the literature^18,31,35,36^. An important success measure for pain interventions comes from the ability of patients to resume their normal lives. MSK pain has been reported as a main driver for loss of productivity^37^, either through presenteeism or absenteeism. In this study, both metrics improved significantly across categories, within the range of the previously reported for other telerehabilitation interventions^38^. Within absenteeism, a recovery rate of one day per week (when considering a full-time job) was observed on SDI category 4 which included those who were socially deprived and located in rural areas. The most socially deprived patients outperformed those in other categories in overall productivity recovery as well as in non-work-related activities. Although we cannot disregard the higher impairment levels at baseline and other moderating factors for this observation, or non-specific effects, these results are nevertheless impressive and can be the effect of a patient-centered DCP. These results support telerehabilitation in light of the $264 billion spent in indirect costs associated with MSK pain in the U.S^39^.

Lower accessibility to in-person physical therapy has been observed among people of color, those with lower education levels and socioeconomic status, and living in rural areas^40,41^, and is considered a contributor to worse outcomes^27^. Moreover, low adherence to physical therapy programs have been associated with poor clinical outcomes^42^. Although telehealth has been touted as a means to address access and adherence barriers, disparities in the adoption of such alternatives related with SDH have been reported^43,44^. Although the COVID-19 pandemic may have impacted the receptivity, compliance and overall perception on the utility of telehealth, especially regarding access to unavailable care options, or by eliminating the need for traveling and in-person contact, opportunities for improvement do remain.

Despite the overall high completion rate observed in this study, which was within the range of the previously reported for real-world in-person physical therapy^30,45^, increased dropout rates were observed across categories, which is also in line with those previously reported^46^.

Considering the lack of consensus in the literature regarding the optimal exercise dosage^47^, current guidelines recommend a personalized approach accounting for each patient’s condition trajectory^6–9^, which was followed herein. High engagement with the DCP was observed based on the number of interactions with the DPT, educational articles read and performed exercise sessions. The number of sessions varied slightly with social deprivation background, with higher SDI categories performing less sessions, which might be explained by a higher proportion of young and overweight patients, as well as the historical association with marginalized groups^43,44,48^. Nevertheless, similar recoveries in all outcomes were observed across SDI categories, suggesting that engagement level was sufficient to promote clinical improvement even amongst those more socially deprived. These improvements were also accompanied by the high satisfaction with the program reported across all categories. The current study design does not permit conclusions about which specific features of the DCP were responsible for the observed outcomes, with potential drivers being the multimodal approach including exercise with real-time biofeedback, education, and CBT, with tailored treatments reflecting clinical and cultural needs. Research shows that when patients feel a sense of trust with their provider, they’re more willing to get the needed care, more likely to adhere to treatment, have fewer symptoms, get more preventive screenings, and ultimately experience better outcomes^49^. Cultural competence is critical to promoting a therapeutic alliance and providing compassionate rapport^50^. One can speculate that the culturally-sensitive clinical team might have contributed to the observed outcomes. Digital interventions supported by optimized communication strategies have been reported to promote similar or even better therapeutic alliance than in-person interventions^51,52^. Communication during digital programs (available chat, video and phone calls) should guarantee not only the practicality of logistics but also ensure respectful and compassionate rapport^52,53^. Ultimately, a MSK pain intervention should leverage therapeutic alliance to empower patients with self-management skills to address their pain and improve quality of life^54^.

Thus, in the light of limited resources and growing demand for rehabilitation services^15^, the results herein reported advocate for scalable digital care delivery systems that acknowledge SDH. However, further controlled and large cohort studies are needed to better characterize the effect of health disparities associated with SDI on digital therapy outcomes. Research should focus on understanding which particular features of a DCP are most impactful on access, engagement and outcomes improvement across different socio-economic contexts. Developing studies designed to better understand the impact of having a culturally competent clinical team on rehabilitation’s success is another high priority. One area ripe for investigation includes developing new integrated approaches featuring digital, in-person and hybrid care models, that can optimize healthcare delivery to the general population, including those most vulnerable. Initiatives such as allowing those without internet to access WiFi hotspots and creationing community hubs where patients can easily access the internet more easily could help in the dissemination of telehealth, and therefore should be further explored. Finally, studies with long-term follow-ups and cost-effectiveness analyses are warranted.

There are several limitations to this study that warrant discussion. First, this study lacked a control group, which precludes the establishment of causality. Second, the program enrolled beneficiaries of employers’ health benefits; therefore, the current cohort may not be representative of the U.S. general population. Third, this study was partially undertaken in the context of the COVID-19 pandemic, which may have impacted perceptions, receptivity and compliance with digital PT programs. Fourth, despite using a socioeconomic metric encompassing seven domains and accounting for additional confounders, we cannot dismiss the possible effects of other moderating factors. Fifth, the study design does not allow for disentangling the impact of each DCP component on outcomes. Lastly, this study lacked long-term follow-up, which would have enabled to ascertain the benefits of the program at later time points across different SDI. This study also presents important strengths, namely the use of a vast real-world cohort from all U.S. states that included the whole range of SDI, thus enhancing generalizability. A second strength is the DCP itself, which includes features designed to mitigate health equity gaps that standard care has struggled to address. A third advantage is the use of validated metrics for both physical and psychological outcomes, which is in contrast to those reported in other digital interventions^55^. Importantly, this study demonstrates that a digital care program designed to promote health equity is feasible and similarly accepted by patients from different socio-economical backgrounds. Finally, the idea of investigating social deprivation within a digital program provides a foundation for future work in this field to identify areas of improvement.

In conclusion, multimodal, patient-centered digital care may represent a solution for addressing health inequities in MSK pain management. This study covers a wide range of SDI indices across the U.S., reporting similar and significant improvements in pain, analgesic consumption, mental health and productivity despite the greater disease burden observed in the socioeconomically vulnerable. This study showcases the potential of digital care to promote health equity, opening new avenues for future research and development.

## Methods

### Study design

This is a post hoc analysis of a decentralized, single-arm investigation into clinical and engagement-related outcomes of patients with musculoskeletal (MSK) conditions following a DCP delivered between June 18th 2020 and August 3rd 2022. The trial was prospectively approved (New England IRB number 120190313) and registered on ClinicalTrials.gov (NCT04092946) on September 17th 2019.

### Population

Adult (≥18 years of age) beneficiaries of employer health plans (employees, spouses and dependents) from all U.S. states (including Washington D.C.) were recruited through a variety of platforms, including the postal system, e-mail, leaflets and posters. Patients reporting chronic MSK pain (persistent or recurring pain for more than 12 weeks in either an ankle, elbow, hip, knee, low back, neck, shoulder, or wrist/hand) were invited to apply to Sword Health’s (Draper, Utah, United States) DCP through a dedicated enrollment website. Exclusion criteria included: (1) a health condition (e.g., cardiac, respiratory) incompatible with at least 20 min of light to moderate exercise; (2) undergoing cancer treatment; and (3) serious neurological signs or symptoms such as neurological weakness, numbness, or bowel or bladder dysfunction. All participants provided electronic informed consent to take part in the study (waiver of documentation of consent approved by New England IRB).

### Intervention

**Fig. 4:**
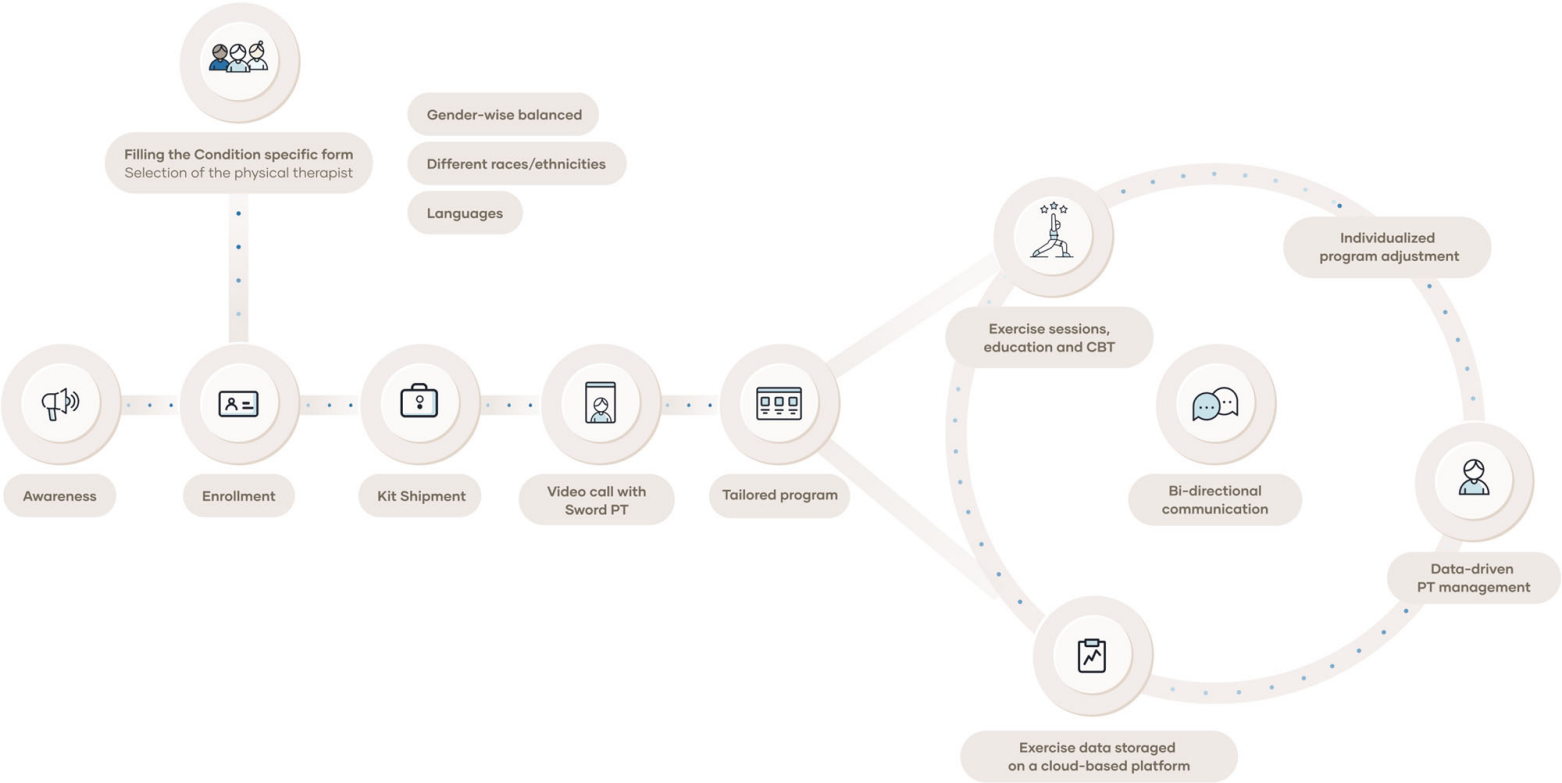
Participant’s journey through the digital care program. At enrollment, each patient is entitled to choose a Doctor of Physical Therapy (DPT) from a diverse pool of physical therapists, in terms of gender, race/ethnicity, cultural background, and spoken languages. The DPT is then responsible for tailoring the program to meet each patient’s needs. The program consists of exercise sessions, education, and cognitive-behavioral therapy (CBT). The data collected from exercise sessions and feedback from patients allow the DPT to continuously adjust the program. Bi-directional communication is available throughout the intervention.

The DCP was composed of exercise, education and CBT administered in a 4-, 8-, or 12-week program depending on each patient’s condition. During enrollment, a baseline condition form was filled out and a video call was performed, triggering the condition-specific shipment of a kit (Fig. 4). The kit consisted of an FDA–listed class II medical device which included a mobile app on a dedicated tablet (that provides real-time video and audio biofeedback on exercise execution through the use of motion trackers or the tablet’s camera), and a cloud-based portal (that enables asynchronous and remote monitoring by the assigned Doctor of Physical Therapy (DPT)). A WiFi hotspot was provided to all participants without internet connection. Each patient was entitled to choose their therapist from a pool of certified DPTs. The clinical team was diverse gender-wise and included professionals from seven race/ethnicities, covering five languages who completed diversity, equity and inclusion training. The selected DPT was responsible for tailoring (according to the initial assessment and continuous monitoring during the study) and adjusting the program (e.g., range of motion, number of exercises, number of sets and repetitions and the type of exercise) to the patient’s specific needs. Exercise performance (namely the range of motion, execution, movement compensations and skipped exercises) and self-reported levels of pain and fatigue during exercise were considered.

Three exercise sessions per week were recommended and performed independently at patients’ convenience. Adherence, existence or absence of movement errors, and level of pain and fatigue during exercises were registered, being used by the DPT to adjust sessions.

Additionally, patients were provided with condition-specific education content and CBT, through written articles, audio content, and interactive modules. These were developed according to current clinical guidelines and prior research^6,7,56,57^, to augment MSK-related health literacy, providing pain self-management skills and to improve mental distress. The CBT program was based on mindfulness, acceptance and commitment therapy and empathy-focused therapy. Bidirectional communication with the DPT was ensured through a built-in secure chat on a smartphone app and video calls. Participants who skipped exercise sessions for 28 consecutive days were considered dropouts. Discharge occurred when (1) goals with the intervention were met; and (2) significant improvements were achieved.

### Social determinants of health (SDH)

A Social Deprivation Index (SDI), based on 2019 data, was assigned to each patient based on their ZIP code^58^. The SDI provides a measure of area-level deprivation as a proxy for SDH by converting seven domains (income, education, employment, housing, household, transportation, age, demographics) into an index score ranging from 1 to 100. Higher scores equate to increased social deprivation.

Additionally, patients were coded to a specific rural-urban commuting area (RUCA) according to their ZIP codes^59^ (urban= 1 to 3 and rural= 4 to 10). To assess the impact of availability of healthcare facilities, each patient’s geo-coordinates were cross-referenced with county-level geographic distribution of healthcare resources (filtered for: clinics, doctors, hospitals and rehabilitation units)^60^ (made available through https://healthsites.io/). The density and proximity of healthcare facilities were calculated considering 6- and 18-miles radii. Additionally, patients’ race and ethnicity were selected choosing from Asian, Black, Hispanic, non-Hispanic white, other and prefer not to specify.

### Clinical outcomes

Assessment surveys collected at baseline, 4-, 8-, and 12-weeks were used to analyze the longitudinal mean changes in outcomes between baseline and program-end. The outcomes included:

1. Numerical Pain Rating Scale (NPRS) specific for the symptomatic body region: “Please rate your average pain over the last 7 days” from 0 (no pain at all) to 10 (worst pain imaginable)”. A Minimum Clinically Important Difference (MCID) was considered to be 30%^61^;
2. Analgesic consumption: “Are you currently taking any pain medication?” (Yes/No);
3. Mental health: Anxiety was assessed by the 7-item Generalized Anxiety Disorder (GAD-7) scale (range 0-21)^62^, and depression was assessed by the 9-item Patient Health Questionnaire (PHQ-9) (range 0-27)^63^, in which higher scores denote worse symptomatology;
4. Work Productivity and Activity Impairment (WPAI) questionnaire collected within employed population to assess overall work impairment (WPAI overall), presenteeism (WPAI work), absenteeism (WPAI time) and activities impairment (WPAI activity)^64^, with higher scores denoting higher impairment;
5. Engagement: measured through the following: A) time spent performing exercise sessions; B) completed exercise sessions; C) sessions per week; D) articles read; E) interactions with the DPT (including video and phone calls, and text messages);
6. Satisfaction through the question: “On a scale from 0 to 10, how likely is it that you would recommend this intervention to a friend or neighbor?”.

### Statistical analysis

The cohort was categorized into fifths using SDI as the indexing variable. Continuous data was tested for normality by calculating skewness and kurtosis. Comparisons between SDI categories for demographic characteristics, clinical outcomes at baseline, and engagement metrics were performed using 1-way ANOVA or Kruskal-Wallis for quantitative variables, or Chi-square test for categorical variables. Bonferroni post-hoc was used to correct for multiple comparisons.

A multiple-group latent growth curve analysis (mLGCA) was used to assess outcome changes across the program (NPRS, GAD-7, PHQ-9, WPAI) following an intention-to-treat analysis. LGCA, widely used in longitudinal studies^65,66^, estimates overall change based on individual trajectories considering time as a continuous variable. This methodology is estimated as a structural equation model^67^, with the advantages of providing a measure of fitness and addressing missing data through full information maximum likelihood (FIML)^68^. FIML outperforms listwise deletion and other imputation models^68^. mLGCA allows for the creation of separate models in different groups, accounting for unbalanced group size, while simultaneously permitting intergroup comparisons. mLGCA estimates all parameters simultaneously precluding the need for multiple comparison corrections. An additional mLGCA analysis focused on individuals with clinically relevant baseline scores was performed: ≥5 points for GAD-7 and PHQ-9^62,63^, and >0 for WPAI (overall, work, time, and activity). The impact of gender, BMI and race/ethnicity was assessed through a conditional model. A robust sandwich estimator was used for standard errors. The impact of the aforementioned covariates plus rurality on training time was assessed by latent basis growth curve analysis.

A latent ordinal regression analysis was performed to longitudinally assess the latent distribution of analgesic consumption from baseline to program-end within and between categories.

A binary variable for reaching the MCID for pain^61^ was created with patients who achieved a reduction of 30% encoded as 1, while all other patients were encoded as 0. The odds ratio (OR) for being a dropout and for reaching the pain MCID at program-end was calculated using binary logistic regression with SDI group as an ordinal variable (up to 4th order polynomial), adjusting for the same previously mentioned covariates.

All statistical analyses were conducted using commercially available software (SPSS v22, IBM, Armonk, NY) and R (version 4.2.2, R Foundation for Statistical Computing), and geocoding using Python (version 3.9.7, Python software foundation). The level of significance was set at *P* < 0.05 for all tests considering a two-sided hypothesis test.

### Reporting summary

Further information on research design is available in the Nature Research Reporting Summary linked to this article.

### Supplementary information

Supplementary Information

Reporting Summary

## Data Availability

The datasets used and/or analyzed during the current study are available from the corresponding author upon reasonable request.

## Code availability

Code for all statistical analyses may be available upon reasonable request from the corresponding author.
