# Supplementary material for: The potential of a multimodal digital care program in addressing healthcare inequities in musculoskeletal pain management
Source: NPJ Digit Med. 2023 Oct 10;6:188. doi: 10.1038/s41746-023-00936-2 (PMC10564877; doi:10.1038/s41746-023-00936-2)
Supplement: Supplementary file 1 — Supplementary Information [file 41746_2023_936_MOESM1_ESM.pdf]

Supplementary Material

The potential of a multimodal digital care program in addressing healthcare inequities in musculoskeletal pain management

Supplementary Table 1. Clinical outcomes at baseline stratified by Social Deprivation Index categories

Clinical Outcome mean (SD)	N	Category 1 (SDI 1-20)	N	Category 2 (SDI 21-40)	N	Category 3 (SDI 41-60)	N	Category 4 (SDI 61-80)	N	Category 5 (SDI 81-100)	p-value[1]
Pain	3666	4.6 (2.0)	2903	4.7 (2.0)	2398	4.8 (2.0)	1874	4.9 (2.0)	1221	5.0 (2.0)	<.001
Medication intake (binary), # (%)	-	792 (21.6)	-	655 (22.6)	-	615 (25.6)	-	451 (24.1)	-	294 (24.1)	.004[2]
GAD-7 ≥ 5	1048	8.3 (3.7)	881	8.7 (4.0)	768	8.9 (4.0)	593	9.3 (4.3)	434	9.5 (4.5)	<.001
GAD-7	3665	2.9 (4.1)	2903	3.2 (4.4)	2398	3.4 (4.5)	1874	3.5 (4.8)	1221	3.9 (5.1)	<.001
PHQ-9 ≥ 5	722	9.0 (4.1)	667	9.12 (4.1)	609	9.3 (4.2)	492	9.8 (4.6)	345	9.7 (4.8)	.006
PHQ-9	3665	2.2 (3.9)	2903	2.5 (4.2)	2398	2.8 (4.4)	1874	2.99 (4.8)	1221	3.2 (4.9)	<.001
WPAI - Overall >0	1640	28.9 (20.3)	1456	30.2 (21.5)	1223	31.1 (22.1)	952	33.1 (23.4)	646	35.4 (23.8)	<.001
WPAI - Overall	3108	15.3 (20.6)	2515	17.5 (22.2)	2021	18.8 (23.0)	1597	19.7 (24.3)	1056	21.6 (25.4)	<.001
WPAI - Work >0	1595	27.0 (17.9)	1405	27.9 (18.5)	1177	28.6 (18.8)	906	30.2 (19.6)	620	31.4 (20.1)	<.001
WPAI - Work	3091	13.9 (18.6)	2490	15.7 (19.6)	1997	16.86 (20.1)	1572	17.4 (21.2)	1040	18.7 (21.9)	<.001
WPAI - Time >0	282	21.0 (26.2)	272	24.1 (29.5)	217	27.2 (31.1)	178	30.3 (33.6)	165	29.1 (31.2)	.006
WPAI - Time	3108	1.9 (9.9)	2515	2.6 (12.2)	2021	2.9 (13.2)	1597	3.4 (14.7)	1056	4.6 (16.2)	<.001
WPAI - Activity >0	2769	33.9 (21.5)	2226	34.7 (21.8)	1857	35.6 (22.1)	1416	36.9 (22.7)	896	38.9 (23.4)	0.005
WPAI - Activity	3665	25.6 (23.7)	2903	26.6 (24.1)	2398	27.6 (24.5)	1874	27.9 (25.4)	1221	28.5 (26.3)	<.001

[1]1-way ANOVA and Pearson Chi-square test

Abbreviations: GAD-7, Generalized Anxiety Disorder 7-item scale; PHQ-9, Patient Health 9-item questionnaire; WPAI, Work Productivity and Activity Impairment questionnaire. Statistically significant p-values were italicized

Supplementary Table 2. Baseline and end program estimated outcome for each SDI category following an intention-to-treat[1]

Outcome	Time	Category 1 (SDI 0-20)	Category 2 (SDI 21-40)	Category 3 (SDI 41-60)	Category 4 (SDI 61-80)	Category 5 (SDI 81-100)
GAD-7	Baseline	2.93 (2.8;3.06)	3.43 (3.25;3.61)	3.31 (3.12;3.49)	3.46 (3.24;3.67)	3.92 (3.63;4.2)
	Program end	1.69 (1.54;1.84)	2.29 (2.05;2.52)	2.16 (1.94;2.39)	2.1 (1.86;2.34)	2.27 (1.97;2.58)
	p-value	*<.001*	*<.001*	*<.001*	*<.001*	*<.001*
	Mean Change	-1.24 (-1.39;-1.08)	-1.14 (-1.37;-0.91)	-1.14 (-1.36;-0.92)	-1.36 (-1.6;-1.12)	-1.64 (-1.95;-1.34)
PHQ-9	Baseline	2.23 (2.11;2.36)	2.82 (2.64;3)	2.73 (2.56;2.91)	2.96 (2.75;3.18)	3.22 (2.95;3.5)
	Program end	1.36 (1.22;1.5)	1.75 (1.54;1.96)	1.65 (1.45;1.85)	1.86 (1.62;2.1)	1.97 (1.67;2.27)
	p-value	*<.001*	*<.001*	*<.001*	*<.001*	*<.001*
	Mean Change	-0.87 (-1.02;-0.73)	-1.07 (-1.29;-0.85)	-1.09 (-1.29;-0.88)	-1.1 (-1.35;-0.86)	-1.25 (-1.55;-0.95)
WPAI - Overall	Baseline	15.16 (14.44;15.88)	18.7 (17.71;19.68)	18.84 (17.84;19.83)	19.6 (18.42;20.77)	21.59 (20.06;23.11)
	Program end	10.03 (8.96;11.1)	11.16 (9.68;12.65)	11.1 (9.67;12.53)	12.02 (10.42;13.63)	11.31 (9.26;13.36)
	p-value	*<.001*	*<.001*	*<.001*	*<.001*	*<.001*
	Mean Change	-5.13 (-6.26;-3.99)	-7.54 (-9.07;-6)	-7.74 (-9.22;-6.25)	-7.57 (-9.29;-5.85)	-10.28 (-12.45;-8.12)
WPAI - Work	Baseline	13.87 (13.22;14.52)	16.8 (15.93;17.67)	16.9 (16.02;17.77)	17.34 (16.32;18.37)	18.73 (17.4;20.05)
	Program end	8.68 (7.73;9.62)	8.72 (7.52;9.91)	8.78 (7.64;9.92)	10.38 (8.98;11.79)	9.72 (8.01;11.44)
	p-value	*<.001*	*<.001*	*<.001*	*<.001*	*<.001*
	Mean Change	-5.19 (-6.2;-4.19)	-8.08 (-9.36;-6.81)	-8.12 (-9.34;-6.9)	-6.96 (-8.47;-5.45)	-9.01 (-10.81;-7.2)
WPAI - Time	Baseline	1.89 (1.55;2.24)	2.88 (2.31;3.44)	2.84 (2.27;3.41)	3.34 (2.62;4.06)	4.55 (3.57;5.52)
	Program end	1.75 (1.17;2.34)	2.97 (1.9;4.05)	2.71 (1.73;3.68)	2.28 (1.4;3.16)	2.13 (0.88;3.38)
	p-value	.669	.867	.800	.056	*.001*
	Mean Change	-0.14 (-0.78;0.5)	0.1 (-1.01;1.2)	-0.13 (-1.16;0.89)	-1.06 (-2.15;0.03)	-2.42 (-3.85;-0.98)

WPAI - Activity	Baseline	25.48 (24.72;26.24)	27.41 (26.44;28.38)	27.81 (26.83;28.8)	27.76 (26.61;28.9)	28.34 (26.87;29.82)
	Program end	14.47 (13.42;15.52)	14.39 (13.04;15.73)	14.32 (12.99;15.64)	15.07 (13.56;16.59)	13.98 (12.16;15.81)
	p-value	*<.001*	*<.001*	*<.001*	*<.001*	*<.001*
	Mean Changes	-11.01 (-12.11;-9.91)	-13.02 (-14.46;-11.58)	-13.5 (-14.93;-12.06)	-12.69 (-14.32;-11.05)	-14.36 (-16.31;-12.41)

[1]Data represents mean (95% Confidence Intervals). *P* values represent comparison between program-end and baseline means performed through multiple-group latent growth curve analysis, with statistically significant *P* values italicized. Abbreviations: GAD-7, Generalized Anxiety Disorder 7-item scale; PHQ-9, Patient Health 9-item questionnaire; WPAI, Work Productivity and Activity Impairment questionnaire.

Supplementary Table 3. Model estimates from Latent Growth Curve analysis stratified by SDI categories: Intention-to-treat[1]

Intention-to-treat

[redacted]

	Intercept - 1-20		Slope - 1-20		Curve - 1-20	
Outcome	Mean (95%CI)	p-value	Mean (95%CI)	p-value	Mean (95%CI)	p-value
Pain Level	4.57 (4.51, 4.63)	< *.001*	-0.38 (-0.41, -0.36)	< *.001*	0.02 (0.02, 0.02)	< *.001*
GAD-7 ≥5	8.29 (8.07, 8.51)	< *.001*	-0.85 (-0.95, -0.75)	< *.001*	0.04 (0.03, 0.05)	< *.001*
GAD-7	2.93 (2.8, 3.06)	< *.001*	-0.21 (-0.24, -0.17)	< *.001*	0.01 (0.01, 0.01)	< *.001*
PHQ-9 ≥5	8.96 (8.66, 9.26)	< *.001*	-1.1 (-1.22, -0.98)	< *.001*	0.06 (0.05, 0.07)	< *.001*
PHQ-9	2.23 (2.11, 2.36)	< *.001*	-0.18 (-0.21, -0.14)	< *.001*	0.01 (0.01, 0.01)	< *.001*
WPAI - Overall >0	28.76 (27.77, 29.74)	< *.001*	-3.14 (-3.57, -2.71)	< *.001*	0.16 (0.13, 0.2)	< *.001*
WPAI - Overall	15.16 (14.44, 15.88)	< *.001*	-1.15 (-1.41, -0.89)	< *.001*	0.06 (0.04, 0.08)	< *.001*
WPAI - Activity >0	33.72 (32.93, 34.52)	< *.001*	-3.45 (-3.75, -3.14)	< *.001*	0.17 (0.15, 0.2)	< *.001*
WPAI - Activity	25.48 (24.72, 26.24)	< *.001*	-2.19 (-2.45, -1.92)	< *.001*	0.11 (0.08, 0.13)	< *.001*
WPAI - Work >0	26.87 (25.99, 27.74)	< *.001*	-3.08 (-3.46, -2.7)	< *.001*	0.16 (0.13, 0.19)	< *.001*
WPAI - Work	13.87 (13.22, 14.52)	< *.001*	-1.1 (-1.33, -0.86)	< *.001*	0.06 (0.04, 0.08)	< *.001*
WPAI - Time >0	20.81 (17.79, 23.84)	< *.001*	-2.9 (-3.9, -1.9)	< *.001*	0.14 (0.06, 0.22)	< *.001*
WPAI - Time	1.89 (1.55, 2.24)	< *.001*	-0.07 (-0.21, 0.07)	0.332	0 (-0.01, 0.02)	0.455

[redacted]

	Diff Intercept vs 1-20		Diff Slope vs 1-20		Diff Curve vs 1-20	
Outcome	Mean (95%CI)	p-value	Mean (95%CI)	p-value	Mean (95%CI)	p-value
Pain Level	0.14 (0.04, 0.23)	*0.004*	-0.03 (-0.07, 0.01)	0.111	0 (0, 0.01)	0.198
GAD-7 ≥5	0.39 (0.05, 0.74)	*0.024*	0.05 (-0.1, 0.19)	0.533	0 (-0.02, 0.01)	0.492
GAD-7	0.29 (0.08, 0.49)	*0.006*	0.03 (-0.03, 0.08)	0.330	0 (-0.01, 0)	0.203

PHQ-9 ≥5	0.15 (-0.29, 0.58)	*0.508*	0.07 (-0.11, 0.25)	0.450	-0.01 (-0.02, 0.01)	0.491
PHQ-9	0.29 (0.09, 0.49)	*0.004*	-0.01 (-0.06, 0.05)	0.767	0 (-0.01, 0)	0.759
WPAI - Overall >0	1.29 (-0.18, 2.76)	*0.086*	-0.16 (-0.79, 0.46)	0.614	0.02 (-0.03, 0.07)	0.475
WPAI - Overall	2.14 (1.02, 3.25)	< *.001*	-0.21 (-0.62, 0.2)	0.313	0.01 (-0.02, 0.05)	0.551
WPAI - Activity >0	0.83 (-0.37, 2.03)	*0.176*	-0.33 (-0.8, 0.13)	0.162	0.03 (-0.01, 0.07)	0.155
WPAI - Activity	1 (-0.15, 2.15)	*0.089*	-0.26 (-0.66, 0.14)	0.200	0.02 (-0.02, 0.05)	0.285
WPAI - Work >0	0.82 (-0.48, 2.12)	*0.216*	-0.09 (-0.65, 0.46)	0.741	0.01 (-0.03, 0.06)	0.553
WPAI - Work	1.71 (0.72, 2.71)	< *.001*	-0.2 (-0.56, 0.16)	0.266	0.01 (-0.02, 0.04)	0.427
WPAI - Time >0	2.96 (-1.62, 7.54)	*0.205*	-1.42 (-2.92, 0.08)	*0.063*	0.11 (0, 0.23)	*0.059*
WPAI - Time	0.67 (0.09, 1.26)	*0.024*	-0.11 (-0.34, 0.13)	0.371	0.01 (-0.01, 0.03)	0.536

[redacted]

	Diff Intercept vs 1-20		Diff Slope vs 1-20		Diff Curve vs 1-20	
Outcome	Mean (95%CI)	p-value	Mean (95%CI)	p-value	Mean (95%CI)	p-value
Pain Level	0.19 (0.09, 0.29)	< *.001*	0 (-0.04, 0.04)	0.991	0 (0, 0)	0.894
GAD-7 ≥5	0.56 (0.21, 0.92)	*0.002*	0.03 (-0.13, 0.18)	0.748	0 (-0.01, 0.01)	0.948
GAD-7	0.5 (0.28, 0.72)	< *.001*	0.02 (-0.05, 0.08)	0.587	0 (-0.01, 0)	0.769
PHQ-9 ≥5	0.29 (-0.15, 0.74)	0.199	0.05 (-0.14, 0.24)	0.622	0 (-0.02, 0.01)	0.752
PHQ-9	0.58 (0.37, 0.8)	< *.001*	-0.02 (-0.09, 0.04)	0.443	0 (0, 0.01)	0.794
WPAI - Overall >0	2.25 (0.68, 3.83)	*0.005*	0.48 (-0.2, 1.17)	0.163	-0.04 (-0.1, 0.01)	0.14
WPAI - Overall	3.54 (2.32, 4.76)	< *.001*	0.03 (-0.41, 0.47)	0.897	-0.02 (-0.06, 0.02)	0.323
WPAI - Activity >0	1.68 (0.41, 2.96)	*0.010*	0.25 (-0.25, 0.76)	0.328	-0.03 (-0.07, 0.01)	0.134
WPAI - Activity	1.93 (0.7, 3.17)	*0.002*	0.11 (-0.32, 0.55)	0.603	-0.02 (-0.06, 0.01)	0.205
WPAI - Work >0	1.64 (0.26, 3.01)	*0.020*	0.29 (-0.31, 0.9)	0.344	-0.03 (-0.09, 0.02)	0.204
WPAI - Work	2.93 (1.84, 4.02)	< *.001*	-0.04 (-0.43, 0.35)	0.826	-0.02 (-0.05, 0.02)	0.333
WPAI - Time >0	6.14 (1.04, 11.24)	*0.018*	-0.21 (-1.74, 1.33)	0.791	0.02 (-0.1, 0.14)	0.792
WPAI - Time	0.98 (0.32, 1.65)	*0.004*	0.12 (-0.14, 0.38)	0.363	-0.01 (-0.03, 0.01)	0.470

[redacted]

	Diff Intercept vs 1-20		Diff Slope vs 1-20		Diff Curve vs 1-20	
Outcome	Mean (95%CI)	p-value	Mean (95%CI)	p-value	Mean (95%CI)	p-value
Pain Level	0.27 (0.16, 0.38)	< *.001*	0.01 (-0.03, 0.06)	0.502	0 (-0.01, 0)	0.395
GAD-7 ≥5	0.99 (0.58, 1.4)	< *.001*	-0.02 (-0.2, 0.16)	0.797	0 (-0.01, 0.02)	0.85
GAD-7	0.53 (0.28, 0.78)	< *.001*	-0.01 (-0.08, 0.06)	0.705	0 (-0.01, 0.01)	0.934
PHQ-9 ≥5	0.8 (0.29, 1.31)	*0.002*	0.1 (-0.13, 0.32)	0.397	-0.01 (-0.03, 0.01)	0.431
PHQ-9	0.73 (0.48, 0.98)	< *.001*	-0.04 (-0.11, 0.03)	0.288	0 (0, 0.01)	0.579
WPAI - Overall >0	4.2 (2.42, 5.98)	< *.001*	0.01 (-0.79, 0.8)	0.985	0 (-0.07, 0.06)	0.911
WPAI - Overall	4.44 (3.06, 5.81)	< *.001*	-0.08 (-0.6, 0.43)	0.752	-0.01 (-0.05, 0.03)	0.65
WPAI - Activity >0	3 (1.57, 4.43)	< *.001*	0.14 (-0.42, 0.7)	0.624	-0.03 (-0.07, 0.02)	0.251
WPAI - Activity	2.28 (0.9, 3.65)	*0.001*	0.2 (-0.27, 0.68)	0.401	-0.03 (-0.07, 0.01)	0.155
WPAI - Work >0	3.17 (1.62, 4.71)	< *.001*	-0.26 (-0.96, 0.44)	0.469	0.02 (-0.04, 0.08)	0.489
WPAI - Work	3.48 (2.26, 4.69)	< *.001*	-0.2 (-0.65, 0.26)	0.395	0 (-0.03, 0.04)	0.833
WPAI - Time >0	9.47 (3.68, 15.26)	*0.001*	-1.22 (-2.9, 0.45)	*0.152*	0.05 (-0.08, 0.17)	0.486
WPAI - Time	1.45 (0.65, 2.24)	< *.001*	0.16 (-0.15, 0.47)	0.326	-0.02 (-0.05, 0.01)	0.146

[redacted]

	Diff Intercept vs 1-20		Diff Slope vs 1-20		Diff Curve vs 1-20	
Outcome	Mean (95%CI)	p-value	Mean (95%CI)	p-value	Mean (95%CI)	p-value
Pain Level	0.41 (0.28, 0.54)	< *.001*	-0.04 (-0.09, 0.01)	0.101	0 (0, 0.01)	0.197
GAD-7 ≥5	1.16 (0.68, 1.64)	< *.001*	-0.19 (-0.38, 0.01)	0.060	0.01 (0, 0.03)	0.085
GAD-7	0.99 (0.68, 1.3)	< *.001*	-0.09 (-0.17, 0)	0.049	0 (0, 0.01)	0.207
PHQ-9 ≥5	0.75 (0.16, 1.35)	*0.013*	0.21 (-0.04, 0.46)	0.106	-0.02 (-0.04, 0.01)	0.145
PHQ-9	0.99 (0.68, 1.29)	< *.001*	-0.03 (-0.1, 0.05)	0.534	0 (-0.01, 0.01)	0.872
WPAI - Overall >0	6.64 (4.56, 8.73)	< *.001*	-0.08 (-0.94, 0.79)	0.858	-0.03 (-0.11, 0.05)	0.461
WPAI - Overall	6.43 (4.74, 8.11)	< *.001*	-0.18 (-0.78, 0.41)	0.548	-0.02 (-0.07, 0.03)	0.431
WPAI - Activity >0	4.96 (3.24, 6.68)	< *.001*	-0.47 (-1.14, 0.2)	0.170	0.01 (-0.05, 0.06)	0.825

WPAI - Activity	2.86 (1.2, 4.52)	< *.001*	0.02 (-0.56, 0.6)	0.938	-0.03 (-0.07, 0.02)	0.295
WPAI - Work >0	4.46 (2.65, 6.27)	< *.001*	-0.48 (-1.27, 0.31)	0.234	0.02 (-0.05, 0.09)	0.523
WPAI - Work	4.86 (3.39, 6.33)	< *.001*	-0.41 (-0.94, 0.11)	0.123	0.01 (-0.04, 0.05)	0.723
WPAI - Time >0	8.55 (2.93, 14.17)	*0.003*	-1.05 (-2.74, 0.65)	0.226	0.05 (-0.07, 0.18)	0.396
WPAI - Time	2.65 (1.62, 3.68)	< *.001*	0.12 (-0.28, 0.52)	0.565	-0.03 (-0.06, 0.01)	0.139

[1]multiple-group latent growth curve analysis

Abbreviations: GAD-7, Generalized Anxiety Disorder 7-item scale; PHQ-9, Patient Health 9-item questionnaire; WPAI, Work Productivity and Activity Impairment Questionnaire. Statistically significant p-values were italicized.

Supplementary Table 4. Model fit for both unfiltered and filtered models: intention-to-treat

Outcome	Chi-sq (df)	*p*	Fit<br>RMSEA	CFI	SRMR
Pain Level	260.35 (5)	< .001	**0.065**	**0.95**	**0.022**
GAD-7 ≥5	56.12 (5)	< .001	**0.052**	**0.97**	**0.019**
GAD-7	26.44 (5)	< .001	**0.019**	**1.00**	**0.005**
PHQ-9 ≥5	52.76 (5)	< .001	**0.058**	**0.97**	**0.021**
PHQ-9	24.73 (5)	< .001	**0.018**	**1.00**	**0.006**
WPAI - Overall >0	35.66 (5)	< .001	**0.032**	**0.98**	**0.014**
WPAI - Overall	13.11 (5)	0.022	**0.012**	**1.00**	**0.006**
WPAI - Activity >0	77.85 (5)	< .001	**0.040**	**0.98**	**0.015**
WPAI - Activity	34.92 (5)	< .001	**0.022**	**0.99**	**0.009**
WPAI - Work >0	40.46 (5)	< .001	**0.035**	**0.98**	**0.016**
WPAI - Work	11.67 (5)	0.040	**0.011**	**1.00**	**0.006**
WPAI - Time Missed >0	14.77 (5)	0.011	**0.042**	**0.98**	**0.018**
WPAI - Time Missed	6.42 (5)	**0.267**	**0.005**	**1.00**	**0.005**

Abbreviations: GAD-7, Generalized Anxiety Disorder 7-item scale; PHQ-9, Patient Health 9-item questionnaire; WPAI, Work Productivity and Activity Impairment Questionnaire.

Note: If a significant chi-square is found for a model, then comparative fit index values (CFI) > .9, or root mean square error of approximation values (RMSEA) < .08, or standardized root mean squared residual values (SRMR) <.05 signify models with acceptable fit.[1,2]

Parameters denoting acceptable fit are presented in bold.

Supplementary Table 5. Medication intake changes per category by program-end

Categories	Proportion of participants consuming medication at program-end	Change in the proportion consuming medication at program-end	Change[1] in SD units (95%CI)	p-value
Category 1 (SDI 0-20)	0.17	-0.031	-0.11 (-0.19, -0.03)	*0.007*
Category 2 (SDI 21-40)	0.17	-0.038	-0.13 (-0.22, -0.05)	*0.003*
Category 3 (SDI 41-60)	0.21	-0.051	-0.17 (-0.27, -0.07)	*0.001*
Category 4 (SDI 61-80)	0.21	-0.041	-0.14 (-0.25, 0.02)	*0.026*
Category 5 (SDI 81-100)	0.18	-0.061	-0.22 (-0.37, -0.063)	*0.006*

[1]Estimate refers to the change of the two categories' mean distribution (taking or not taking medication) between baseline and treatment end per category, i.e., to what extent the mean distribution will shift towards not consuming medication. Comparisons were performed through latent ordinal regression analysis. Statistically significant p-values were italicized.

Supplementary Table 6. Impact of covariates in clinical outcomes per Social Deprivation Index (SDI) category

A. Pain

		Intercept					Slope				
Covariate		C1 (SDI 1-20)	C2 (SDI 21-40)	C3 (SDI 41-60)	C4 (SDI 61-80)	C5 (SDI 81-100)	C1 (SDI 1-20)	C2 (SDI 21-40)	C3 (SDI 41-60)	C4 (SDI 61-80)	C5 (SDI 81-100)
Gender = women	mean difference	0.53 (0.4, 0.65)	0.48 (0.33, 0.62)	0.29 (0.13, 0.44)	0.22 (0.04, 0.4)	0.59 (0.37, 0.81)	-0.03 (-0.08, 0.02)	0.01 (-0.05, 0.06)	0.01 (-0.05, 0.08)	0.0 (-0.08, 0.07)	-0.07 (-0.17, 0.03)
	p-value	**< .001**	**< .001**	**< .001**	**0.019**	**0.019**	0.183	0.744	0.687	0.926	0.147
BMI	mean difference	0.04 (0.03, 0.05)	0.03 (0.02, 0.04)	0.04 (0.02, 0.05)	0.04 (0.03, 0.05)	0.04 (0.02, 0.05)	0.0 (0.0, 0.01)	0.0 (0.0, 0.0)	0.0 (0.0, 0.01)	0.0 (0.0, 0.01)	0.0 (-0.01, 0.01)
	p-value	**< .001**	**< .001**	**< .001**	**< .001**	**< .001**	0.321	0.921	0.189	0.556	0.649
Race/Ethnicity											
Non-Hispanic White= reference	mean difference	4.27 (4.16, 4.38)	4.43 (4.31, 4.56)	4.47 (4.33, 4.61)	4.55 (4.38, 4.72)	4.3 (4.08, 4.52)	-0.35 (-0.39, -0.31)	-0.43 (-0.48, -0.38)	-0.39 (-0.45, -0.33)	-0.32 (-0.39, -0.26)	-0.36 (-0.46, -0.26)
	p-value	**< .001**	**< .001**	**< .001**	**< .001**	**< .001**	***< .001***	***< .001***	***< .001***	***< .001***	***< .001***
Asian	mean difference	0.40 (0.17, 0.64)	0.00 (-0.27, 0.27)	0.11 (-0.23, 0.45)	0.00 (-0.36, 0.37)	-0.45 (-0.98, 0.07)	-0.07 (-0.16, 0.03)	-0.04 (-0.16, 0.08)	0.04 (-0.11, 0.19)	-0.07 (-0.21, 0.08)	0.08 (-0.16, 0.33)
	p-value	**< .001**	1.00	0.516	0.983	0.092	0.165	0.515	0.607	0.383	0.509
Black	mean difference	0.25 (-0.09, 0.6)	0.19 (-0.16, 0.54)	0.74 (0.44, 1.05)	0.58 (0.26, 0.91)	0.85 (0.55, 1.16)	-0.03 (-0.19, 0.14)	-0.01 (-0.18, 0.15)	-0.02 (-0.19, 0.15)	-0.07 (-0.23, 0.08)	-0.17 (-0.31, -0.03)
	p-value	**0.153**	**0.292**	**< .001**	**< .001**	**< .001**	0.759	0.873	0.801	0.353	***0.021***

Hispanic	mean difference	0.55 (0.24, 0.86)	0.4 (0.1, 0.69)	0.69 (0.4, 0.98)	0.6 (0.27, 0.93)	0.67 (0.36, 0.98)	-0.08 (-0.2, 0.04)	0.05 (-0.07, 0.18)	0.02 (-0.12, 0.17)	-0.12 (-0.25, 0.0)	0.01 (-0.13, 0.14)
	p-value	**< .001**	**0.010**	**< .001**	**< .001**	**< .001**	0.184	0.398	0.757	0.052	**< .001**
Undisclosed	mean difference	-0.14 (-0.28, 0.01)	-0.17 (-0.33, 0.0)	-0.01 (-0.2, 0.17)	0.03 (-0.19, 0.25)	0.08 (-0.22, 0.37)	0.01 (-0.05, 0.06)	0.05 (-0.01, 0.11)	0.02 (-0.06, 0.09)	-0.03 (-0.11, ´0.06)	0.02 (-0.1, 0.14)
	p-value	0.061	0.051	0.873	0.814	0.613	0.824	0.132	0.674	0.510	0.757
Other	mean difference	0.02 (-0.45, 0.49)	-0.12 (-0.65, 0.42)	-0.16 (-0.59, 0.28)	0.2 (-0.36, 0.76)	0.28 (-0.44, 1.0)	-0.03 (-0.23, 0.17)	0.12 (-0.06, 0.3)	-0.13 (-0.3, 0.05)	-0.1 (-0.41, 0.2)	0.11 (-0.12, 0.34)
	p-value	0.937	0.668	0.487	0.484	0.439	0.774	0.198	0.165	0.505	0.344

Comparisons were performed through a conditional multiple-group latent growth curve analysis.
Abbreviations: C, Category; GAD-7, Generalized Anxiety Disorder 7-item scale; PHQ-9, Patient Health 9-item questionnaire; WPAI, Work Productivity and Activity Impairment questionnaire. Statistically significant p-values were in bold

B. Anxiety

Covariate		Intercept					Slope				
		C1 (SDI 1-20)	C2 (SDI 21-40)	C3 (SDI 41-60)	C4 (SDI 61-80)	C5 (SDI 81-100)	C1 (SDI 1-20)	C2 (SDI 21-40)	C3 (SDI 41-60)	C4 (SDI 61-80)	C5 (SDI 81-100)
Gender = women	mean difference	1.09 (0.84, 1.35)	0.75 (0.43, 1.07)	1.17 (0.82, 1.52)	1.34 (0.92, 1.76)	1.23 (0.67, 1.8)	-0.08 (-0.15, -0.01)	-0.05 (-0.13, 0.04)	0.03 (-0.07, 0.13)	-0.16 (-0.28, -0.05)	0.01 (-0.15, 0.17)
	p-value	**< .001**	**< .001**	**< .001**	**< .001**	**< .001**	0.025	0.269	0.620	0.005	0.913
BMI	mean difference	0.04 (0.02, 0.06)	0.06 (0.03, 0.08)	0.02 (-0.01, 0.05)	0.03 (0.0, 0.06)	0.04 (-0.01, 0.08)	0.0 (-0.01, 0.0)	0.0 (-0.01, 0.01)	0.0 (-0.01, 0.01)	-0.01 (-0.02, 0.0)	0.0 (-0.02, 0.01)
	p-value	**< .001**	**< .001**	0.130	0.060	0.085	0.256	0.659	0.475	0.136	0.603

Race/Ethnicity											
Non-Hispanic Whites = reference	mean difference	2.22 (2.0, 2.43)	2.75 (2.47, 3.03)	2.52 (2.22, 2.81)	2.68 (2.29, 3.07)	3.04 (2.46, 3.63)	-0.15 (-0.21, -0.09)	-0.12 (-0.2, -0.04)	-0.1 (-0.19, -0.02)	-0.07 (-0.18, 0.03)	-0.31 (-0.48, -0.14)
	p-value	**< .001**	**< .001**	**< .001**	**< .001**	**< .001**	**< .001**	**0.003**	**0.021**	0.170	**< .001**
Asian	mean difference	-0.3 (-0.73, 0.13)	-0.6 (-1.09, -0.11)	0.17 (-0.59, 0.94)	-0.52 (-1.28, 0.25)	0.34 (-1.02, 1.69)	0.01 (-0.11, 0.13)	0.0 (-0.16, 0.15)	-0.21 (-0.44, 0.02)	-0.05 (-0.28, 0.18)	0.31 (-0.11, 0.72)
	p-value	0.170	**0.017**	0.656	0.186	0.626	0.886	0.954	0.077	0.675	0.145
Black	mean difference	-0.50 (-1.16, 0.16)	-0.28 (-1.06, 0.51)	-0.54 (-1.24, 0.17)	-0.67 (-1.38, 0.04)	-0.76 (-1.52, 0.0)	0.16 (-0.07, 0.4)	-0.03 (-0.27, 0.21)	0.16 (-0.06, 0.38)	0.01 (-0.19, 0.21)	0.17 (-0.06, 0.4)
	p-value	0.140	0.493	0.134	0.063	0.051	0.180	0.810	0.158	0.921	0.149
Hispanic	mean difference	0.37 (-0.27, 1.02)	0.88 (0.04, 1.72)	1.33 (0.55, 2.11)	-0.06 (-0.84, 0.71)	0.59 (-0.32, 1.5)	0.11 (-0.08, 0.3)	0.0 (-0.20, 0.20)	-0.16 (-0.36, 0.03)	-0.03 (-0.23, 0.16)	-0.12 (-0.36, 0.12)
	p-value	0.258	**0.041**	**< .001**	0.873	0.206	0.248	0.983	0.103	0.730	0.331
Undisclosed	mean difference	0.59 (0.28, 0.89)	0.13 (-0.24, 0.49)	0.47 (0.06, 0.89)	0.38 (-0.16, 0.92)	0.39 (-0.34, 1.13)	-0.10 (-0.18, -0.01)	-0.13 (-0.24, -0.03)	-0.31 (-0.42, -0.19)	-0.17 (-0.32, -0.03)	-0.03 (-0.23, 0.18)
	p-value	**< .001**	0.492	**0.024**	0.172	0.294	**0.022**	**0.010**	**< .001**	**0.019**	0.797
Other	mean difference	0.14 (-1.18, 1.46)	0.20 (-0.85, 1.25)	1.05 (-0.2, 2.3)	0.49 (-0.82, 1.81)	0.93 (-1.05, 2.9)	0.14 (-0.12, 0.41)	0.23 (-0.09, 0.55)	-0.18 (-0.66, 0.3)	-0.14 (-0.45, 0.17)	-0.35 (-0.83, 0.12)
	p-value	0.837	0.711	0.101	0.460	0.359	0.291	0.157	0.472	0.384	0.145

Comparisons were performed through a conditional multiple-group latent growth curve analysis.

Abbreviations: C, Category; GAD-7, Generalized Anxiety Disorder 7-item scale; PHQ-9, Patient Health 9-item questionnaire; WPAI, Work Productivity and Activity Impairment questionnaire. Statistically significant p-values were in bold

C. Depression

Covariate		Intercept C1 (SDI 1-20)	Intercept C2 (SDI 21-40)	Intercept C3 (SDI 41-60)	Intercept C4 (SDI 61-80)	Intercept C5 (SDI 81-100)	Slope C1 (SDI 1-20)	Slope C2 (SDI 21-40)	Slope C3 (SDI 41-60)	Slope C4 (SDI 61-80)	Slope C5 (SDI 81-100)
Gender = women	mean difference	0.48 (0.23, 0.73)	0.42 (0.11, 0.72)	0.74 (0.4, 1.09)	0.93 (0.52, 1.35)	0.82 (0.28, 1.37)	-0.03 (-0.09, 0.04)	-0.05 (-0.14, 0.03)	-0.05 (-0.15, 0.05)	-0.12 (-0.23, 0.0)	-0.07 (-0.21, 0.07)
	p-value	**< .001**	**0.007**	**< .001**	**< .001**	**< .001**	0.430	0.202	0.310	0.042	0.350
BMI	mean difference	0.11 (0.08, 0.13)	0.1 (0.07, 0.12)	0.08 (0.05, 0.11)	0.09 (0.06, 0.12)	0.12 (0.08, 0.17)	-0.01 (-0.02, 0.0)	-0.01 (-0.02, 0.0)	-0.01 (-0.02, 0.0)	-0.01 (-0.02, 0.0)	-0.02 (-0.03, 0.0)
	p-value	**< .001**	**< .001**	**< .001**	**< .001**	**< .001**	**0.011**	**0.012**	0.152	0.189	**0.010**
Race/Ethnicity											
Non-Hispanic Whites = reference	mean difference	1.76 (1.55, 1.96)	2.09 (1.82, 2.37)	2.15 (1.85, 2.45)	2.26 (1.87, 2.66)	2.52 (1.94, 3.1)	-0.12 (-0.18, -0.06)	-0.07 (-0.14, 0.01)	-0.09 (-0.18, 0.0)	-0.08 (-0.18, 0.02)	-0.12 (-0.29, 0.04)
	p-value	**< .001**	**< .001**	**< .001**	**< .001**	**< .001**	**< .001**	0.070	**0.049**	0.132	0.139
Asian	mean difference	-0.13 (-0.51, 0.26)	-0.34 (-0.78, 0.10)	0.0 (-0.69, 0.68)	-0.65 (-1.32, 0.01)	0.67 (-0.58, 1.92)	0.02 (-0.09, 0.13)	-0.10 (-0.24, 0.04)	-0.11 (-0.4, 0.18)	0.10 (-0.07, 0.27)	0.08 (-0.22, 0.37)
	p-value	0.520	0.130	0.989	0.053	0.296	0.740	0.172	0.453	0.265	0.606
Black	mean difference	0.0 (-0.72, 0.72)	-0.05 (-0.73, 0.62)	-0.08 (-0.86, 0.69)	-0.43 (-1.13, 0.27)	-0.6 (-1.33, 0.14)	0.11 (-0.16, 0.37)	-0.12 (-0.35, 0.11)	0.17 (-0.09, 0.42)	-0.09 (-0.31, 0.14)	0.17 (-0.04, 0.38)
	p-value	0.994	0.876	0.840	0.230	0.110	0.426	0.300	0.195	0.454	0.120
Hispanic	mean difference	0.0 (-0.59, 0.6)	0.7 (-0.14, 1.54)	0.10 (-0.55, 0.74)	0.11 (-0.68, 0.89)	0.12 (-0.76, 1.0)	0.02 (-0.14, 0.18)	-0.08 (-0.28, 0.12)	0.0 (-0.23, 0.23)	0.01 (-0.2, 0.23)	0.03 (-0.18, 0.25)

	p-value	0.990	0.104	0.767	0.789	0.790	0.795	0.456	0.985	0.894	0.778
Undisclosed	mean difference	1.03 (0.74, 1.33)	0.67 (0.33, 1.02)	0.77 (0.37, 1.18)	0.69 (0.16, 1.21)	0.58 (-0.14, 1.3)	-0.19 (-0.27, -0.11)	-0.28 (-0.38, -0.18)	-0.29 (-0.4, -0.17)	-0.24 (-0.39, -0.09)	-0.24 (-0.43, -0.05)
	p-value	**< .001**	**< .001**	**< .001**	**0.011**	0.117	**< .001**	**< .001**	**< .001**	**0.002**	**0.016**
Other	mean difference	0.49 (-0.9, 1.87)	0.72 (-0.45, 1.88)	0.66 (-0.69, 2.01)	-0.12 (-1.47, 1.24)	0.59 (-1.56, 2.73)	0.15 (-0.25, 0.55)	-0.01 (-0.37, 0.34)	-0.27 (-0.69, 0.15)	0.08 (-0.35, 0.5)	0.0 (-0.31, 0.31)
	p-value	0.491	0.226	0.336	0.867	0.591	0.469	0.934	0.211	0.730	0.992

Comparisons were performed through a conditional multiple-group latent growth curve analysis.
Abbreviations: C, Category; GAD-7, Generalized Anxiety Disorder 7-item scale; PHQ-9, Patient Health 9-item questionnaire; WPAI, Work Productivity and Activity Impairment questionnaire. Statistically significant p-values were in bold

D. WPAI - Overall

		Intercept					Slope				
Covariate		C1 (SDI 1-20)	C2 (SDI 21-40)	C3 (SDI 41-60)	C4 (SDI 61-80)	C5 (SDI 81-100)	C1 (SDI 1-20)	C2 (SDI 21-40)	C3 (SDI 41-60)	C4 (SDI 61-80)	C5 (SDI 81-100)
Gender = women	mean difference	4.28 (2.86, 5.7)	2.99 (1.29, 4.69)	2.26 (0.31, 4.21)	1.15 (-1.21, 3.51)	3.38 (0.32, 6.44)	-0.24 (-0.75, 0.27)	-0.19 (-0.82, 0.45)	0.63 (-0.08, 1.34)	-0.12 (-1.01, 0.78)	-1.11 (-2.19, -0.02)
	P-value	**< .001**	**< .001**	**0.020**	0.340	0.340	0.348	0.563	0.083	0.800	0.045
BMI	mean difference	0.32 (0.19, 0.44)	0.24 (0.09, 0.38)	0.43 (0.27, 0.58)	0.36 (0.18, 0.54)	0.35 (0.13, 0.56)	0.0 (-0.05, 0.05)	0.01 (-0.04, 0.06)	-0.05 (-0.10, 0.0)	-0.04 (-0.11, 0.03)	0.0 (-0.09, 0.09)
	P-value	**< .001**	**0.001**	**< .001**	**< .001**	**< .001**	0.996	0.664	0.076	0.250	0.957
Race/Ethnicity											

Non-Hispanic Whites = reference	mean difference	13.16 (11.95, 14.37)	15.26 (13.8, 16.73)	15.99 (14.29, 17.69)	18.32 (16.2, 20.45)	17.62 (14.76, 20.49)	-1.12 (-1.54, -0.7)	-1.03 (-1.62, -0.45)	-1.34 (-2.01, -0.68)	-0.73 (-1.6, 0.14)	-0.57 (-1.77, 0.63)
	P-value	**< .001**	**< .001**	**< .001**	**< .001**	**< .001**	**< .001**	**< .001**	**< .001**	0.099	0.344
Asian	mean difference	0.72 (-1.65, 3.1)	-1.16 (-4.08, 1.77)	3.36 (-0.43, 7.14)	2.1 (-2.54, 6.74)	-0.79 (-6.68, 5.11)	-0.2 (-1.13, 0.73)	-0.05 (-1.17, 1.07)	-1.44 (-2.73, -0.15)	-0.17 (-1.94, 1.6)	-1.3 (-3.15, 0.54)
	P-value	0.550	0.440	0.082	0.374	0.794	0.672	0.930	0.028	0.852	0.165
Black	mean difference	2.43 (-1.98, 6.84)	1.39 (-2.72, 5.49)	6.55 (1.96, 11.14)	0.71 (-3.53, 4.96)	4.57 (0.02, 9.11)	0.56 (-1.56, 2.68)	-0.8 (-2.54, 0.94)	0.03 (-1.96, 2.02)	0.42 (-1.34, 2.17)	-0.42 (-2.02, 1.19)
	P-value	0.281	0.508	**0.005**	0.740	**0.049**	0.605	0.368	0.977	0.641	0.611
Hispanic	mean difference	1.24 (-2.54, 5.02)	-0.41 (-4.13, 3.32)	6.0 (2.04, 9.96)	3.09 (-1.5, 7.68)	3.94 (-0.79, 8.68)	1.16 (-0.71, 3.03)	0.16 (-1.07, 1.38)	0.55 (-1.21, 2.3)	-1.52 (-3.18, 0.14)	-0.14 (-1.96, 1.68)
	P-value	0.520	0.830	**0.003**	0.190	0.103	0.223	0.804	0.542	0.073	0.881
Undisclosed	mean difference	-1.32 (-2.96, 0.32)	0.92 (-1.19, 3.03)	0.72 (-1.65, 3.08)	-0.46 (-3.31, 2.39)	-0.05 (-3.87, 3.77)	-0.03 (-0.57, 0.51)	-0.75 (-1.48, -0.03)	-0.21 (-1.01, 0.6)	-1.02 (-2.05, 0.02)	0.35 (-1.02, 1.72)
	P-value	0.114	0.394	0.553	0.752	0.979	0.906	**0.041**	0.616	0.054	0.614
Other	mean difference	6.58 (-0.53, 13.69)	6.15 (-0.64, 12.95)	-2.56 (-8.54, 3.42)	0.92 (-6.02, 7.87)	5.78 (-3.85, 15.41)	0.98 (-0.8, 2.75)	-0.84 (-3.85, 2.17)	0.73 (-1.94, 3.41)	-0.39 (-2.5, 1.72)	-0.26 (-2.53, 2.0)
	p-value	0.070	0.076	0.402	0.794	0.240	0.281	0.586	0.591	0.718	0.820

Comparisons were performed through a conditional multiple-group latent growth curve analysis.
Abbreviations: C, Category; GAD-7, Generalized Anxiety Disorder 7-item scale; PHQ-9, Patient Health 9-item questionnaire; WPAI, Work Productivity and Activity Impairment questionnaire. Statistically significant p-values were in bold.

E. WPAI - Activity

Covariate		Intercept C1 (SDI 1-20)	Intercept C2 (SDI 21-40)	Intercept C3 (SDI 41-60)	Intercept C4 (SDI 61-80)	Intercept C5 (SDI 81-100)	Slope C1 (SDI 1-20)	Slope C2 (SDI 21-40)	Slope C3 (SDI 41-60)	Slope C4 (SDI 61-80)	Slope C5 (SDI 81-100)
Gender = women	mean difference	5.21 (3.71, 6.7)	4.48 (2.78, 6.18)	3.7 (1.79, 5.61)	3.73 (1.45, 6.0)	6.4 (3.47, 9.33)	-0.28 (-0.8, 0.24)	0.02 (-0.58, 0.62)	0.24 (-0.44, 0.92)	0.46 (-0.33, 1.25)	-0.81 (-1.88, 0.26)
	p-value	**< .001**	**< .001**	**< .001**	**0.001**	**0.001**	0.293	0.949	0.483	0.250	0.138
BMI	mean difference	0.56 (0.44, 0.68)	0.50 (0.36, 0.63)	0.55 (0.41, 0.7)	0.62 (0.44, 0.79)	0.54 (0.33, 0.75)	-0.01 (-0.06, 0.03)	-0.02 (-0.06, 0.03)	-0.01 (-0.06, 0.04)	-0.06 (-0.12, 0.0)	-0.07 (-0.16, 0.01)
	p-value	**< .001**	**< .001**	**< .001**	**< .001**	**< .001**	0.561	0.536	0.625	**0.049**	0.099
Race/Ethnicity											
Non-Hispanic Whites = reference	mean difference	23.95 (22.66, 25.25)	25.72 (24.23, 27.21)	25.97 (24.25, 27.69)	25.85 (23.78, 27.92)	24.35 (21.4, 27.29)	-2.20 (-2.65, -1.76)	-2.57 (-3.14, -2.01)	-2.18 (-2.81, -1.55)	-1.76 (-2.5, -1.02)	-1.35 (-2.56, -0.13)
	P-value	**< .001**	**< .001**	**< .001**	**< .001**	**< .001**	**< .001**	**< .001**	**< .001**	**< .001**	**0.030**
Asian	mean difference	-1.33 (-3.94, 1.27)	-5.67 (-8.79, -2.56)	-1.90 (-5.98, 2.19)	-0.74 (-5.15, 3.67)	-3.74 (-10.05, 2.56)	-0.03 (-1.06, 1.0)	0.69 (-0.4, 1.77)	-1.01 (-2.40, 0.37)	-1.36 (-2.99, 0.26)	-0.55 (-2.83, 1.73)
	P-value	0.320	**< .001**	0.362	0.742	0.245	0.956	0.214	0.150	0.100	0.636
Black	mean difference	-4.34 (-8.54, -0.15)	-5.52 (-9.48, -1.57)	-0.45 (-4.8, 3.90)	-2.04 (-6.11, 2.03)	-0.14 (-4.49, 4.22)	0.82 (-1.0, 2.64)	-0.55 (-2.2, 1.10)	1.01 (-0.83, 2.84)	-0.86 (-2.35, 0.63)	-0.96 (-2.53, 0.6)
	P-value	**0.043**	**0.006**	0.840	0.330	0.950	0.376	0.510	0.281	0.259	0.228
Hispanic	mean difference	1.56 (-2.42, 5.54)	-2.66 (-6.48, 1.16)	1.16 (-2.57, 4.89)	-2.68 (-6.77, 1.4)	2.91 (-1.75, 7.57)	-0.08 (-1.46, 1.3)	0.98 (-0.36, 2.33)	0.46 (-0.89, 1.81)	-0.89 (-2.33, 0.54)	-0.28 (-1.87, 1.31)

	P-value	0.443	0.173	0.541	0.198	0.221	0.911	0.152	0.504	0.223	0.732
Undisclosed	mean difference	-2.88 (-4.61, -1.15)	-3.8 (-5.82, -1.79)	-2.8 (-5.02, -0.57)	-0.82 (-3.56, 1.92)	-3.09 (-6.73, 0.54)	0.54 (-0.03, 1.12)	-0.01 (-0.68, 0.65)	-0.2 (-0.97, 0.57)	-0.74 (-1.64, 0.16)	0.2 (-1.17, 1.58)
	P-value	**0.001**	**< .001**	**0.014**	0.557	0.095	0.064	0.973	0.611	0.108	0.770
Other	mean difference	4.73 (-2.16, 11.62)	2.53 (-3.86, 8.93)	-1.79 (-8.35, 4.77)	7.4 (-0.78, 15.59)	2.35 (-5.9, 10.59)	-1.45 (-3.55, 0.65)	-0.35 (-2.69, 1.98)	0.45 (-2.47, 3.37)	-1.23 (-4.47, 2.02)	-1.02 (-4.1, 2.07)
	P-value	0.178	0.437	0.593	0.076	0.577	0.176	0.767	0.763	0.460	0.518

Comparisons were performed through a conditional multiple-group latent growth curve analysis.
Abbreviations: C, Category; GAD-7, Generalized Anxiety Disorder 7-item scale; PHQ-9, Patient Health 9-item questionnaire; WPAI, Work Productivity and Activity Impairment questionnaire. Statistically significant p-values were in bold

Supplementary Table 7. Impact of covariates in total training time per Social Deprivation Index category

Covariate		Intercept					Slope				
		C1 (SDI 1-20)	C2 (SDI 21-40)	C3 (SDI 41-60)	C4 (SDI 61-80)	C5 (SDI 81-100)	C1 (SDI 1-20)	C2 (SDI 21-40)	C3 (SDI 41-60)	C4 (SDI 61-80)	C5 (SDI 81-100)
Race/Ethnicity											
Non-Hispanic Whites = reference	mean difference	63.25 (61.1, 65.4)	62.67 (60.07, 65.26)	61.45 (58.62 ,64.27)	62.92 (59.49, 66.36)	59.74 (55.67, 63.81)	19.06 (16, 22.11)	21.89 (18.57, 25.2)	21.55 (17.66, 25.43)	21.93 (17.3, 26.56)	23.17 (16.9, 29.43)
	p-value	*< .001*	*< .001*	*< .001*	*< .001*	*< .001*	*< .001*	*< .001*	*< .001*	*< .001*	*< .001*
Asian	mean difference	-3.27 (-7.47, 0.92)	-3.88 (-8.68, 0.93)	-2.93 (-9.48, 3.63)	-1.88 (-8.1, 4.34)	1.34 (-7.79, 10.47)	-0.74 (-3.35, 1.87)	0.57 (-3.03, 4.17)	-6.29 (-10.92, -1.67)	0.06 (-4.91, 5.03)	6.47 (-3.64, 16.58)
	p-value	0.126	0.114	0.381	0.553	0.774	0.576	0.758	*0.008*	0.982	0.210
Black	mean difference	-8.08 (-13.83, -2.33)	-0.63 (-6.12, 4.85)	-4 (-9.62, 1.62)	0.28 (-5.47, 6.02)	-2.62 (-7.62, 2.38)	-5.63 (-8.97, -2.29)	-2.21 (-6.17, 1.75)	-2.35 (-7.10, 2.40)	-1.45 (-6.11, 3.21)	-0.37 (-5.88, 5.14)
	p-value	*0.006*	0.821	0.163	0.925	0.304	*0.001*	0.274	0.332	0.542	0.896
Hispanic	mean difference	-8.35 (-13.66 ,-3.03)	-1.8 (-6.94, 3.35)	-9.29 (-14.18, -4.39)	-4.84 (-9.64, -0.03)	-0.51 (-5.54, 4.53)	-3.51 (-6.52 ,-0.49)	-2.45 (-6.43, 1.53)	-7.02 (-10.58 ,-3.46)	-2.83 (-6.99, 1.34)	-0.2 (-5.48, 5.08)
	p-value	*0.002*	0.494	*< .001*	*0.049*	0.844	*0.023*	*0.227*	*< .001*	*0.184*	0.941
Undisclosed	mean difference	20.27 (17.17, 23.37)	18.59 (14.98, 22.19)	18.53 (14.49 ,22.57)	19.04 (14.34, 23.74)	21.34 (15.34, 27.35)	9.29 (7.03, 11.56)	12.07 (8.71, 15.43)	11.5 (7.98, 15.02)	12.81 (8.37, 17.26)	19.79 (11.01, 28.57)
	p-value	*< .001*	*< .001*	*< .001*	*< .001*	*< .001*	*< .001*	*< .001*	*< .001*	*< .001*	*< .001*
Other	mean difference	-1.46 (-10.81, 7.9)	-6.53 (-14.24, 1.17)	-5.57 (-15.3, 4.17)	-8.34 (-17.16, 0.49)	5.88 (-8.23, 19.99)	-6.4 (-11.54, -1.26)	-5.14 (-9.43, -0.86)	-3.74 (-11.9, 4.42)	-6.1 (-13.56, 1.36)	-8.69 (-18.01, 0.62)

	p-value	0.760	0.097	0.263	0.064	0.414	*0.015*	*0.019*	0.369	0.109	0.067
Rurality = Rural	mean difference	2.51 (-3.83, 8.86)	7.59 (3.05, 12.13)	1.41 (-2.72, 5.53)	3.01 (-1.93, 7.95)	-3.47 (-10.24, 3.31)	2.16 (-1.14, 5.46)	4.58 (1.22, 7.95)	3.69 (0.56, 6.82)	3.63 (-0.17, 7.43)	2.65 (-4.62, 9.91)
	p-value	0.437	*0.001*	0.503	0.232	0.316	0.199	*0.008*	*0.021*	0.061	0.476
Gender = women	mean difference	-6.53 (-9.01, -4.06)	-6.93 (-9.74, -4.12)	-3.66 (-6.77, -0.56)	-8.67 (-12.15, -5.19)	-8.01 (-12.26, -3.76)	-1.76 (-3.28, -0.24)	-2.39 (-4.45, -0.33)	-0.13 (-2.45, 2.20)	-2.83 (-5.7, 0.04)	-1.94 (-6.43, 2.56)
	p-value	< *.001*	< *.001*	*0.021*	< *.001*	< *.001*	*0.023*	*0.023*	0.915	0.053	0.399
BMI	mean difference	-0.71 (-0.90, -0.52)	-0.62 (-0.82, -0.43)	-0.67 (-0.87, -0.47)	-0.54 (-0.79, -0.28)	-0.35 (-0.64, -0.05)	-0.27 (-0.4, -0.15)	-0.35 (-0.51, -0.2)	-0.53 (-0.71, -0.35)	-0.42 (-0.64, -0.2)	-0.35 (-0.66, -0.04)
	p-value	< *.001*	< *.001*	< *.001*	< *.001*	*0.022*	< *.001*	< *.001*	< *.001*	< *.001*	*0.025*

Comparisons were performed through a latent basis growth curve analysis.
Abbreviations: C, Category; GAD-7, Generalized Anxiety Disorder 7-item scale; PHQ-9, Patient Health 9-item questionnaire; WPAI, Work Productivity and Activity Impairment questionnaire. Statistically significant p-values were italicized.

REFERENCES

1. Iacobucci D. Structural equations modeling: Fit Indices, sample size, and advanced topics. *Journal of Consumer Psychology*. 2010;20(1):90-98. doi:10.1016/j.jcps.2009.09.003
2. Brown TA. *Confirmatory factor analysis for applied research*. Confirmatory factor analysis for applied research. The Guilford Press; 2006:xiii, 475-xiii, 475.
