# Supplementary material for: The potential of a multimodal digital care program in addressing healthcare inequities in musculoskeletal pain management
Source: NPJ Digit Med. 2023 Oct 10;6:188. doi: 10.1038/s41746-023-00936-2 (PMC10564877; doi:10.1038/s41746-023-00936-2)
Supplement: Supplementary file 2 — Reporting Summary [file 41746_2023_936_MOESM2_ESM.pdf]

nature portfolio

Corresponding author(s): Fabíola Costa

Last updated by author(s): Sep 18, 2023

Reporting Summary

Nature Portfolio wishes to improve the reproducibility of the work that we publish. This form provides structure for consistency and transparency in reporting. For further information on Nature Portfolio policies, see our Editorial Policies and the Editorial Policy Checklist.

Statistics

For all statistical analyses, confirm that the following items are present in the figure legend, table legend, main text, or Methods section.

n/a	Confirmed	
☐	☒	The exact sample size (*n*) for each experimental group/condition, given as a discrete number and unit of measurement
☐	☒	A statement on whether measurements were taken from distinct samples or whether the same sample was measured repeatedly
☐	☒	The statistical test(s) used AND whether they are one- or two-sided<br>*Only common tests should be described solely by name; describe more complex techniques in the Methods section.*
☐	☒	A description of all covariates tested
☐	☒	A description of any assumptions or corrections, such as tests of normality and adjustment for multiple comparisons
☐	☒	A full description of the statistical parameters including central tendency (e.g. means) or other basic estimates (e.g. regression coefficient) AND variation (e.g. standard deviation) or associated estimates of uncertainty (e.g. confidence intervals)
☐	☒	For null hypothesis testing, the test statistic (e.g. *F*, *t*, *r*) with confidence intervals, effect sizes, degrees of freedom and *P* value noted<br>*Give P values as exact values whenever suitable.*
☒	☐	For Bayesian analysis, information on the choice of priors and Markov chain Monte Carlo settings
☒	☐	For hierarchical and complex designs, identification of the appropriate level for tests and full reporting of outcomes
☒	☐	Estimates of effect sizes (e.g. Cohen's *d*, Pearson's *r*), indicating how they were calculated

Our web collection on statistics for biologists contains articles on many of the points above.

Software and code

Policy information about availability of computer code

Data collection	Collected data was stored in a database. The networks where Sword Health's infrastructure is hosted are maintained by Google and are included in Google Cloud Platform's SOC2 and HITRUST certifications.
Data analysis	Data analyses were conducted using commercially available software (SPSS v22, IBM, Armonk, NY) and R (version 4.2.2, R Foundation for Statistical Computing), and geocoding using Python (version 3.9.7, Python software foundation).

For manuscripts utilizing custom algorithms or software that are central to the research but not yet described in published literature, software must be made available to editors and reviewers. We strongly encourage code deposition in a community repository (e.g. GitHub). See the Nature Portfolio guidelines for submitting code & software for further information.

Data

Policy information about availability of data

All manuscripts must include a data availability statement. This statement should provide the following information, where applicable:

- Accession codes, unique identifiers, or web links for publicly available datasets
- A description of any restrictions on data availability
- For clinical datasets or third party data, please ensure that the statement adheres to our policy

All relevant data is included in the article or available as supplementary material. The datasets used and/or analysed during the current study are available from the corresponding author on reasonable request.

Research involving human participants, their data, or biological material

Policy information about studies with human participants or human data. See also policy information about sex, gender (identity/presentation), and sexual orientation and race, ethnicity and racism.

Reporting on sex and gender	For study purpose and design, gender was determined based on self-reporting and reported accordingly in the manuscript. This information was collected at a baseline questionnaire. Consent was obtained from participants for sharing of individual-level data. This study included 6935 women and 5080 men. This proportion is in accordance with the higher worldwide prevalence of chronic musculoskeletal conditions among women compared to men found in a systematic analysis of the Global Burden of Disease study 2019 (Journal of orthopaedic translation. 2022;32:49-58).<br>Statistical analysis was performed using gender as a covariate, including a conditional model to assess its impact on outcomes.
Reporting on race, ethnicity, or other socially relevant groupings	Race characterisation was self-reported from participants through the question "Which race of the following best describes you? Please select one answer.". The available categories for selection were "Asian", "Black", "Hispanic", "Non-Hispanic White", "Multi-racial", "Native", "A race/ethnicity not listed here" and "Prefer not to answer".<br>The definition of these categories was based on the guidelines provided by the U.S. Office of Management and Budget (OMB) used by The Census Bureau.<br>Statistical analysis was performed using race as a covariate, including a conditional model to assess its impact on outcomes.<br>Socieconomic context was assessed using social deprivation index (SDI) as a proxy. SDI was developed by Butler et al. using U.S. Census Bureau data. Statistical analyses were performed across SDI categories.
Population characteristics	The following baseline population characteristics were collected: age, gender, body mass index, race, education level, employment status, geographic location, and baseline pain intensity, mental health and work productivity impairment.
Recruitment	Adult beneficiaries of employer health plans (employees, spouses and dependents) from all U.S. states (including Washington D.C.) were recruited through a variety of platforms, including the postal system, e-mail, leaflets and posters.
Ethics oversight	The trial was prospectively approved by the New England Institutional Review Board (number 120190313).

Note that full information on the approval of the study protocol must also be provided in the manuscript.

Field-specific reporting

Please select the one below that is the best fit for your research. If you are not sure, read the appropriate sections before making your selection.

☒ Life sciences ☐ Behavioural & social sciences ☐ Ecological, evolutionary & environmental sciences

For a reference copy of the document with all sections, see nature.com/documents/nr-reporting-summary-flat.pdf

Life sciences study design

All studies must disclose on these points even when the disclosure is negative.

Sample size	This single-arm, intervention study was conducted in an estimated enrollment period of 5 years, aiming to include as many participants as possible over this period. For this reason, sample size estimates were not computed, given that the target number of individuals enrolled (N= 12,062) greatly exceeds the number of individuals that would need to be included to detect meaningful clinical change in a pre-post analysis (which range between 60 and 300 individuals, according to sample size estimates from other studies).
Data exclusions	Patients with no Zip Codes available
Replication	This is a real-world study, conducted with patients beneficiaries of employer health plans (employees, spouses and dependents) from all U.S. states (including Washington D.C.) that included the whole range of social deprivation index, denoting a diverse population in socioeconomic status.
Randomization	This is a post hoc analysis of a single-arm investigation and, therefore, randomization was not performed.
Blinding	Not applicable.

Reporting for specific materials, systems and methods

We require information from authors about some types of materials, experimental systems and methods used in many studies. Here, indicate whether each material, system or method listed is relevant to your study. If you are not sure if a list item applies to your research, read the appropriate section before selecting a response.

Materials & experimental systems

n/a	Involved in the study	
☒	☐	Antibodies
☒	☐	Eukaryotic cell lines
☒	☐	Palaeontology and archaeology
☒	☐	Animals and other organisms
☐	☒	Clinical data
☒	☐	Dual use research of concern
☒	☐	Plants

Methods

n/a	Involved in the study	
☒	☐	ChIP-seq
☒	☐	Flow cytometry
☒	☐	MRI-based neuroimaging

Clinical data

Policy information about clinical studies

All manuscripts should comply with the ICMJE guidelines for publication of clinical research and a completed CONSORT checklist must be included with all submissions.

Clinical trial registration	NCT04092946
Study protocol	The study protocol is available from the corresponding author on reasonable request.
Data collection	Collected data was stored in a database. The networks where SWORD Health's infrastructure is hosted are maintained by Google and are included in Google Cloud Platform's SOC2 and HITRUST certifications.
Outcomes	The outcomes included:<br>- Pain: self-reported by a 11-point numerical pain rating scale;<br>- Analgesic consumption: assessed by the question "Are you taking any pain medication? Yes/No";<br>- Anxiety symptoms: self-reported by Generalized Anxiety Discorder 7-item scale;<br>- Depression symptoms: self-reported by the Patient Health 9-item Questionnaire;<br>- Work productivity impairment: self-reported by the Work Productivity and Activity Impairment for General Health questionnaire, version 2.0;<br>- Patient's engagement: comprising time spent performing exercise sessions, completed exercise sessions, sessions per week, articles read and interactions with the physical therapist (including video and phone calls, and text mesages). Exercise data was objectively collected by the device, and the data concerning articles read and interactions was automatically collected by the smartphone app;<br>- Patient's satisfaction: assessed through the question: "On a scale from 0 to 10, how likely is it that you would recommend this intervention to a friend or neighbour?".
